# Mobile Network Softwarization: Technological Foundations and Impact on Improving Network Energy Efficiency

**DOI:** 10.3390/s26020503

**Published:** 2026-01-12

**Authors:** Josip Lorincz, Amar Kukuruzović, Dinko Begušić

**Affiliations:** 1 Faculty of Electrical Engineering, Mechanical Engineering and Naval Architecture (FESB), University of Split, R. Boškovića 32, 21000 Split, Croatia; 2 Croatian Academy of Engineering (HATZ), Kačićeva 28, 10000 Zagreb, Croatia

**Keywords:** NFV, SDN, energy efficiency, power, 5G, B5G, 6G, mobile network, AI, VNF, cloud, programmability, optimization, MNO, RAN, controller, virtualization, core, SON

## Abstract

This paper provides a comprehensive overview of mobile network softwarization, emphasizing the technological foundations and its transformative impact on the energy efficiency of modern and future mobile networks. In the paper, a detailed analysis of communication concepts known as software-defined networking (SDN) and network function virtualization (NFV) is presented, with a description of their architectural principles, operational mechanisms, and the associated interfaces and management frameworks that enable programmability, virtualization, and centralized control in modern mobile networks. The study further explores the role of cloud computing, virtualization platforms, distributed SDN controllers, and resource orchestration systems, outlining how they collectively support mobile network scalability, automation, and service agility. To assess the maturity and evolution of mobile network softwarization, the paper reviews contemporary research directions, including SDN security, machine-learning-assisted traffic management, dynamic service function chaining, virtual network function (VNF) placement and migration, blockchain-based trust mechanisms, and artificial intelligence (AI)-enabled self-optimization. The analysis also evaluates the relationship between mobile network softwarization and energy consumption, presenting the main SDN- and NFV-based techniques that contribute to reducing mobile network power usage, such as traffic-aware control, rule placement optimization, end-host-aware strategies, VNF consolidation, and dynamic resource scaling. Findings indicate that although fifth-generation (5G) mobile network standalone deployments capable of fully exploiting softwarization remain limited, softwarized SDN/NFV-based architectures provide measurable benefits in reducing network operational costs and improving energy efficiency, especially when combined with AI-driven automation. The paper concludes that mobile network softwarization represents an essential enabler for sustainable 5G and future beyond-5G systems, while highlighting the need for continued research into scalable automation, interoperable architectures, and energy-efficient softwarized network designs.

## 1. Introduction

The softwarization of mobile networks presents one of the key directions in the modernization and advancement of network infrastructures, significantly changing the way communication services are delivered and managed. This approach entails a shift from traditional hardware-defined solutions to flexible software-defined systems. Such a model enables mobile network operators (MNOs) to respond quickly and efficiently to the growing demands for faster, more reliable, and scalable communication services.

A key aspect of softwarization is an approach known as software-defined networking (SDN), which enables centralized management of network resources through software controllers. The SDN architecture separates the control layer (plane) from the data layer forwarding layer (plane), allowing dynamic management of network resources and real-time adaptation of network policies. This enhances network flexibility and agility, reduces the need for physical interventions, and enables faster deployment of new services [1,2].

Another important element of mobile network softwarization is an approach known as network function virtualization (NFV), which enables the virtualization of network functions in devices such as routers, switches, and firewalls. NFV allows these functions to run on standardized servers instead of specialized hardware devices. This technology reduces hardware costs and enables simpler and more flexible management of network functions, improving network efficiency and scalability [2,3].

Cloud computing also has a significant role in the softwarization of mobile networks. The use of cloud technologies allows the distribution of network functionalities and services in the cloud infrastructure, enabling users to access advanced services such as security solutions, big data processing, and advanced data analytics. Cloud infrastructure allows operators to provide scalable and reliable services, reducing the time required for the implementation of new functionalities and enhancing the end-user experience [2,4].

The standardization of softwarization is also crucial for the successful implementation of these technologies in the realization of future mobile networks. Standardization organizations such as ETSI (European Telecommunications Standards Institute), ONF (Open Networking Foundation), and ITU (International Telecommunication Union) play a key role in defining standards and protocols that ensure interoperability and compatibility of various network solutions exploiting cloud infrastructure, and SDN and NFV services.

This paper examines the technological aspects of softwarization related to SDN and NFV technologies and the standardization process of these technologies. The objective is to provide an overview of the current state and future possibilities of softwarization in mobile networks, highlighting its advantages, challenges, and impact on the industry. Additionally, the paper considers how softwarization of mobile networks can contribute to improving mobile network energy efficiency.

### 1.1. The Importance of Mobile Network Softwarization

The softwarization of mobile networks is one of the most important approaches to the development and advancement of modern network infrastructures, bringing numerous advantages that transform the way communication services are delivered and managed. Its significance can be observed through several key aspects, including increased flexibility and agility, reduced operational costs, faster innovation and competitiveness, improved security and network resilience, support for advanced services and technologies, and improvement in network energy efficiency.

Softwarization enables operators to adapt network resources and functionalities according to current user and market demands. SDN and NFV facilitate the rapid introduction of new services, modification of network policies, and performance optimization without the need for significant physical interventions. This flexibility is particularly important in a dynamic environment with evolving user requirements [5].

The transition from specialized hardware equipment to standardized server infrastructures significantly reduces initial and operational costs. The NFV enables the consolidation of various network services to a single platform, reducing maintenance and network management costs. Additionally, software solutions facilitate the automation of many operational processes, which further decreases labor costs [6,7]. Additionally, softwarization accelerates the innovation cycle, allowing for faster testing and deployment of new features and services. This is particularly important in the telecommunications industry, where competition and technological advancements are highly intense. With the increasing level of softwarization in mobile networks, operators can respond more quickly to market and user demands, which enlarges their competitiveness and adaptability to new technologies and trends [8]. Also, mobile network softwarization solutions enable the implementation of advanced security functions and policies that can be rapidly adjusted to cyber threats and attacks. Centralized network management provides better monitoring and faster response to security incidents. Additionally, NFV enhances network resilience to physical failures and enables faster recovery from unexpected outages [9,10,11].

Softwarization is essential for enabling technologies like fifth-generation (5G) mobile networks or beyond 5G networks (B5G) and different advanced services such as Internet of Things (IoT), virtual and extended reality (VR and XR), autonomous driving, smart everything (agriculture, health, industry, cities, …), etc. These technologies require high flexibility, scalability, and network efficiency, which can only be achieved through SDN architectures. Softwarization allows operators to offer advanced services, such as personalized network solutions, big data processing, and customized security functions [12,13,14]. Additionally, the softwarization of mobile networks provides significantly better approaches to addressing network energy efficiency issues, as this is one of the key factors related to the operational expenditures (OPEX) of network operators [15].

### 1.2. Future Perspectives of Mobile Network Softwarization

The full potential of mobile network softwarization is expected to be manifested with the implementation of 5G mobile networks, since there was a low possibility for network softwarization in fourth-generation (4G) and previous mobile network generations. With the introduction of 5G networks, in addition to the expected higher network capacities, the need for ensuring diverse services that cannot be easily implemented while retaining existing mobile network architectures emerged [16].

**Figure 1:**
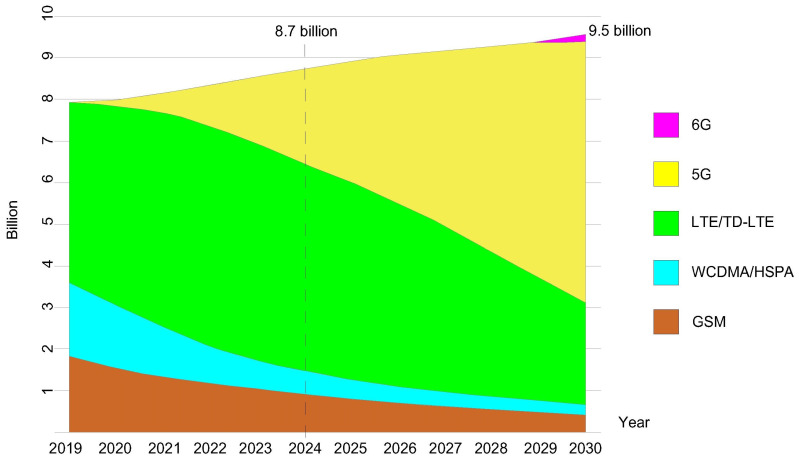
Mobile subscriptions by technology [17].

**Figure 2:**
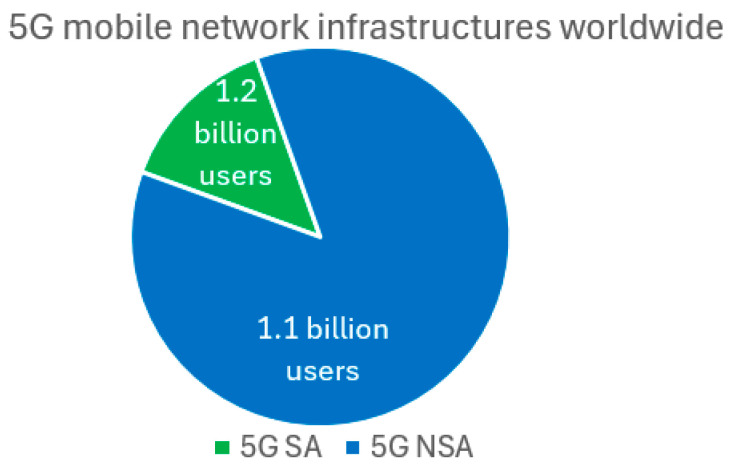
The distribution of users in the global implementations of the 5G SA and 5G NSA infrastructure in 2024.

In Figure 1, the trends until 2030 in the variation in the number of global users in different active mobile network generation technologies are presented [17]. According to Figure 1, by 2030, the number of 5G mobile network users within the standalone (SA) and non-standalone (NSA) 5G network implementations will dominate in the global distribution of users in mobile networks. The difference between these two 5G network types is that 5G NSA still relies on the 4G network infrastructure, which is not fully applicable for the realization of full network softwarization, as SA 5G network architectures are. According to document [17], the number of 5G mobile network users in 2024 is estimated to be 2.3 billion. Additionally, Figure 2 illustrates the percentage ratio of implemented 5G SA and 5G NSA infrastructure on the global level with the corresponding number of users in 2024. The number of 5G SA users is approximately 1.2 billion, accounting for about 50% of the total users relying on 5G infrastructure. On the other hand, according to [18], only 12% of the total implemented 5G infrastructure is 5G SA-based infrastructure that can fully apply the concept of network softwarization. Thus, the full potential of mobile network softwarization in 5G and B5G networks is yet to be exploited, especially because the users are highly interested in the 5G and B5G network services, which realization is currently only feasible through the implementation of mobile network softwarization.

According to [19], projections indicate that by 2028, 75% of 5G users will use 5G SA mobile networks. Also, it is estimated that around 60% of 4G subscribers will be converged on 5G core networks within 5G SA networks. Additionally, according to [20], by the 3rd quarter of 2024, only 63 worldwide mobile network operators (MNOs) have commercially implemented 5G networks with SA in a total of 35 countries, thereby applying some form of mobile network softwarization. The economic value of the market for key enablers of network softwarization (which includes SDN and NFV technologies) equals 38.34 billion USD in 2024, and is growing at an annual rate of 16.4% which is expected to reach 130.76 billion USD by 2032 [21].

The presented 5G SA network deployments suggest that the implementation of 5G SA mobile networks, and consequently the process of mobile network softwarization, is still in its early stages. This contrasts with the intensive research conducted by the academic community in this field, which is already shifting towards the 6G mobile networks.

The increased adoption of 5G SA networks, and thus mobile network softwarization, is crucial for overcoming the stagnation of the mobile services market, which has emerged due to the limited number of implemented 5G SA networks [19]. Given the growing user demand for advanced services based on softwarization, it is evident that massive technological development of mobile network softwarization in the 5G network implementation is still at the very beginning. However, market interest indicates its inevitable expansion in the near future. Thus, a significant increase in the implementation of 5G SA, 5G NSA, and B5G networks worldwide is expected in the coming years, which will result in an increase in mobile networks’ energy consumption [22,23,24], while on the other hand, it will drive further softwarization of network services and enable their broader application across various industries.

This paper provides an insight into the softwarization of mobile networks, with a particular focus on technological aspects, standardization, and the impact of softwarization on energy efficiency. The contribution lies in analyzing key technologies such as SDN and NFV, considering their technical characteristics, advantages, and challenges. Furthermore, the study explores the role of standardization in achieving interoperability and harmonizing network solutions, referencing relevant organizations and standards. Additionally, it examines current and future techniques for energy savings, as well as the energy efficiency improvements enabled by the softwarization of mobile networks. The paper highlights the importance of softwarization in the future development of mobile networks and its significance for MNOs, emphasizing its contribution to energy efficiency. Since energy efficiency becomes a central objective of future telecommunications systems, the paper also emphasizes the need for deeper integration of AI-driven automation in achieving mobile network sustainability.

The rest of the paper is organized as follows: Section 2 outlines the key features of SDN/NFV concepts, which include recent security and optimization solutions, edge and blockchain applications in NFV, and AI-driven automation and energy efficiency. Section 3 describes the data plane, the control plane, and the application plane of SDN and three parts of the NFV concept that include hardware, virtualization, and the virtual layer of NFV infrastructure (NFVI), components and lifecycle management interfaces of the VNF layer, and the ETSI Management and Orchestration framework. In Section 4, the impact of softwarization on the energy efficiency of mobile networks is analyzed, quantifying the share of energy costs and presenting the main mechanisms for reducing network energy consumption through the implementation of advanced SDN/NFV architectures. In Section 5, the concluding remarks are presented, emphasizing that the transition to SDN/NFV-based networks critically enhances the energy-efficiency, flexibility, scalability, and resource utilization of mobile networks, while reducing operational costs through virtualization and centralized orchestration.

## 2. Background on Mobile Network Softwarization

One of the main reasons for introducing softwarization in mobile networks is the need for greater flexibility and the ability to rapidly adapt networks to the growing demands of new services. Traditional, mostly hardware-based mobile network infrastructures have often been inflexible and costly to update, limiting MNOs’ ability to innovate and quickly respond to market changes [25]. Additionally, traditional network setup methods relying on the manual configuration of specialized devices are often inefficient, prone to errors, and do not maximize the potential of physical network infrastructure [26]. The introduction of NFV and SDN has enabled the separation of network functions from specialized hardware, allowing MNOs to deploy new services more quickly and easily [27]. By utilizing cloud-adapted architectures, such as microservices architectures based on container implementation, MNOs can achieve greater scalability and cost reduction [25,27]. This transition from traditional to service-based architecture (SBA), for example, in the core part of 5G networks, is an excellent example of how softwarization is transforming this sector of mobile communication networks [25].

The integration of Artificial Intelligence (AI) into mobile network systems has further accelerated the process of network softwarization. AI technologies are used to enhance network management, improve user experience, and optimize business processes [28,29]. For example, AI-driven analytical tools enable MNOs to predict user churn, better manage network resources, and enhance network service quality [30]. The authors in [31] presented how user churn in 5G networks leads to mobile network reconfiguration that also impacts network energy efficiency. Thus, the implementation of AI-driven analytical tools can ultimately help MNOs to reduce maintenance costs and service prices. Real-time processing of large volumes of data realized through network softwarization enables MNOs to make informed decisions and adjust their offerings to meet growing user demands [32]. Such a data-driven approach is crucial in an increasingly demanding market environment.

Massive implementation of IoT-based services will also have in the future a significant role in contributing to the softwarization of mobile networks. As more devices become interconnected, MNOs are increasingly adopting IoT platforms for managing and analyzing data generated by IoT devices [33,34]. Through mobile network softwarization, the adoption of IoT technologies enables MNOs to offer new services, such as smart home solutions and industrial IoT applications, thereby expanding their service portfolios and revenue streams. The convergence of IoT and telecommunications creates new business models and opportunities for innovation, which MNOs seek to capitalize on due to the growing demand for connected services [35]. Furthermore, the softwarization of mobile networks impacts competitive dynamics within the industry. As MNOs adopt cloud computing and software-driven solutions, they start to have a better position to compete with over-the-top (OTT) service providers, who have often disrupted traditional telecommunications revenue streams [36]. By adopting flexible and scalable softwarized network architectures, MNOs can offer innovative services that compete with those of OTT providers, fostering customer loyalty and maintaining market share. This competitive pressure has led to an increased focus on strategic agility, as MNOs must continuously adapt to technological advancements and evolving consumer preferences.

The softwarization of mobile networks also presents challenges that must not be overlooked. As mobile networks become increasingly dependent on software, MNOs must address issues related to security, interoperability, and the management of various execution environments. Ensuring the reliability and performance of software applications across different platforms and devices is crucial to maintaining service quality and customer satisfaction [37]. Additionally, the transition to software-driven models requires significant investments in workforce education and training to adapt to new technologies and methods. Thus, the implementation of mobile network softwarization is a complex process that involves technological advancements, organizational changes, and adaptation to market conditions. Nevertheless, as the telecommunications industry continues to evolve, the ability to integrate software innovations will become essential for operators seeking to remain competitive in this increasingly demanding market.

### 2.1. The Main Characteristic of SDN and NFV Networks

As previously stated, the foundation of softwarization is the use of SDN and NFV for the realization of different network services. The SDN and NFV are interconnected technologies, where SDN focuses on network control, while NFV is designed for the virtualization of network functions. These two technologies are often used together to make the network more flexible, agile, and efficient.

**Figure 3:**
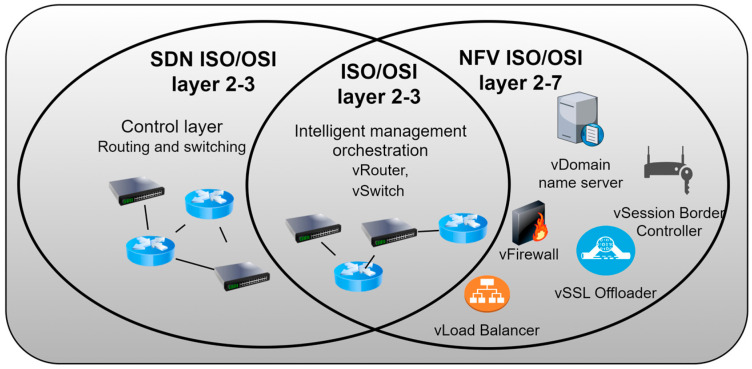
Operation of NFV and SDN technologies on ISO/OSI layers.

Figure 3 illustrates the correlation between NFV and SDN technologies within the context of the ISO/OSI reference model, with an emphasis on the specific layers on which these technologies operate.

NFV encompasses a broader scope of applications within the OSI model, as it is utilized across the second (data link) layer up to the seventh (application) layer. This enables the virtualization of various network functions, including those from higher layers such as virtualized DNS (domain name server), firewalls, and application services (such as virtualized SBC (session border controller), SSL (secure sockets layer) offloader, load balancer, etc.). In contrast, SDN is primarily implemented within the second and third layers, where it facilitates programmable network traffic management through control of switching and routing. Although SDN and NFV can be implemented independently at OSI Layers 2 and 3, a synergistic effect is achieved when these two technologies are deployed in conjunction (Figure 3). A representative example includes virtual switches (vSwitches) and virtual routers (vRouters), where SDN contributes by managing network traffic and routing paths through centralized control mechanisms, while NFV is responsible for the virtualization and instantiation of these network elements.

Given that SDN and NFV are not identical technologies, nor do they share the same foundational objectives or design paradigms, this example illustrates, in a simplified manner in Figure 3, the interrelationship between them within a virtualized network architecture. In order to achieve integrated functionality between SDN and NFV, there must be some form of orchestration or exchange of information to coordinate the operation of SDN and NFV, and consequently, the entire network. This exchange of information occurs through an orchestration interface that connects two entities, the SDN controller and the NFV orchestrator [38]. The NFV orchestrator is an entity that has management and orchestration mechanisms exclusively within the NFV environment. The SDN can have various implementation scenarios and roles within the NFV architectural framework, covering the resource, control, and management aspects of SDN [38]. According to [39], Table 1 shows some of the advantages of implementing NFV technology.

**Table 1:** Main characteristics and advantages of NFV technology [39].

NFV Characteristics	Description
Cost savings	NFV enables network operators to reduce costs by consolidating network functions onto a smaller number of physical devices, thereby lowering hardware and maintenance expenses.
Flexibility and scalability	NFV allows network operators to easily scale network functions as needed, enabling rapid adaptation to changing user demands and market conditions.
Service agility	NFV enables network operators to quickly introduce and innovate new network services, reducing the time required to enter the market and improving competitiveness.
Multi-tenancy support	NFV supports multi-tenancy, allowing multiple users or applications to share the same virtualized infrastructure while maintaining their own isolated and secure network functions.
Reduced complexity	NFV simplifies network operations by enabling network operators to implement, manage, and orchestrate network functions using software, which reduces the need for specialized hardware.

In addition to the advantages listed in Table 1, ETSI in [40] mentions additional benefits of NFV, such as improved return on investment from new services, openness to the market of virtual devices, participants focused solely on software solutions, and the ability to test and deploy new innovative services with lower risk. 

**Table 2:** Advantages of using SDN compared to traditional infrastructures [41].

SDN Characteristics	Description
Energy management	Energy optimization can be applied to various components of the SDN architecture, or SDN can be used as a means for achieving energy savings, either algorithmically or through hardware improvements.
Direct programmability	The network is directly programmable as control functions are separated from forwarding functions, allowing network configuration through proprietary or open-source automation tools.
Centralized management	Network intelligence is logically centralized within the SDN controller, which maintains a global view of the network, presenting it to applications and policies as a single logical switch.
Reduction of CAPEX	Reduces the need to purchase dedicated hardware based on application-specific integrated circuits (ASICs), as new applications can be easily installed on the controller in the application layer.
Reduction of OPEX	Enables algorithmic management of network elements such as hardware or software switches/routers that are programmable, which facilitates improved network implementation, management, and scaling.
Agility and flexibility	Allows organizations to quickly deploy new applications, services, and infrastructure to rapidly adapt to changing business goals.
Enabling innovation	New types of services, applications, and business models can be created directly in the application layer, without disrupting other parts of the network.
Network management and resource utilization	By separating the control and data planes, the system gains flexibility, and resources can be utilized as needed by programming the SDN control or data plane (layer).
Bandwidth	SDN can efficiently utilize available bandwidth by dynamically allocating it according to user needs.

Also, Table 2 presents the advantages of using SDN compared to traditional networks, which are currently predominantly implemented in practice. According to Table 2, the necessity of transitioning from traditional to SDN-type networks is evident for those MNOs that aim to remain competitive in the market, accelerate innovation processes, reduce costs, and become more energy efficient. Table 3 presents the requirements and challenges confronting these two technologies in the process of implementing SDN and NFV.

**Table 3:** Requirements and challenges in the realization of SDN and NFV technology [42].

Characteristics	SDN	NFV
Control	Standardization of control interfaces. Protection of commercial business operational schemes. Measures to avoid performance degradation. Maintaining control of network information for big data development.	Seamless control and service delivery. Real-time and dynamic service provisioning. Creation of granular network policies. Maintaining virtualization information for big data development.
Reliability	Seamless connectivity and fast link recovery. Security requirements in EPC and RAN. Security and reliability of transport and data networks. Balance between performance, security, and flexibility.	High complexity of 5G (technologies, devices, IoT). Seamless and high-quality connectivity. Virtualization of terminal endpoints. Security issues (same physical medium).
Scalability	Support for heterogeneity of technology and devices. Controller messages with performance and survivability (low packet loss levels). Optimization of flow rules and network slicing.	Scalability at the operator level and robustness. Deployment acceleration. Openness and interoperability, global reach, and mutual administration.
Cost- effectiveness	Ability to support a commercial pay-per-service model. Replacement of hardware with software applications. Implementation and procurement of standard network switches (replacing outdated hardware). Shorter time to market and lower implementation risks.	Reduction in energy consumption. Improvement of operational efficiency. Higher capital expenditures. Higher operational costs (short lifecycle of configuration tools).

### 2.2. Research on the Development of SDN

One of the key research areas within SDN is the development of security solutions for managing vulnerabilities arising from its architecture. For example, researchers in [43] analyze an intrusion detection system based on risk analysis, specifically designed for SDN environments, emphasizing the importance of preventive security measures to mitigate potential threats. Similarly, researchers in [44] explore the use of virtualized network functions for identifying and mitigating security threats, which highlights the role of SDN in enhancing network resilience against attacks. These studies underscore the need for implementing robust security frameworks within SDN to protect networks from increasingly complex cyber threats.

Another key aspect of SDN research is the optimization of network performance through the implementation of advanced routing algorithms and traffic management techniques. In [45], a multi-service routing algorithm adapted for spatial information networks is presented, demonstrating how SDN can enhance routing efficiency and reduce latency. The research on applying machine learning techniques for detecting Distributed Denial-of-Service (DDoS) attacks within SDN, showcasing the potential for intelligent traffic management and anomaly detection, is presented in [46]. These improvements illustrate SDN’s capability not only to enhance operational efficiency but also to adaptively respond to cybersecurity network challenges in real-time.

In addition to security and performance optimization, the concept of network slicing (NS) has emerged as a key research area within SDN. NS enables the creation of multiple virtual networks on a single physical infrastructure, each tailored to specific service requirements. This capability is particularly important in 5G networks, where different applications require varying levels of bandwidth, latency, and reliability [47]. The study [48] provides a comprehensive overview of NS using SDN and NFV, outlining the challenges and future directions for this technology. The ability to dynamically allocate resources across different slices enhances the efficiency and adaptability of network services.

### 2.3. Research on the Development of NFV

One of the important research directions is the improvement of the security aspects of NFV. The virtualization of network functions introduces new security vulnerabilities that need to be detected and described for each specific NFV implementation, in order to ensure the integrity and reliability of network services. The authors of [49] present a comprehensive review of anomaly detection based on machine learning in NFV, highlighting various techniques for identifying and mitigating security threats within virtualized environments. The need for robust security frameworks is crucial, as the increasing complexity of networks makes them more susceptible to attacks.

Additionally, NFV is being explored in the context of edge computing, where deploying Virtual Network Functions (VNFs) at the network edge can significantly reduce latency and improve service delivery for applications requiring real-time processing. The study in [50] illustrates how NFV can be utilized in cloud-edge computing environments to optimize service chain deployment, thereby enhancing resource utilization efficiency. This is particularly important for applications in the Internet of Things (IoT) and smart cities, where low latency and high reliability are essential. Furthermore, the researchers in this study introduced an algorithm that impacts resource utilization efficiency, achieving energy consumption reductions of 8.35%, 12.23%, 29.54%, and 52.29% compared to the well-known optimization techniques called Binary Grey Wolf Optimization (BGWO), Double Deep Reinforcement Learning (DDRL), behavior–interaction–priorities (BIP), and mixed integer linear programming (MILP), respectively.

Research on NFV also addresses challenges related to the orchestration of VNFs. Efficient orchestration is crucial for managing the lifecycle of VNFs, including their deployment, scaling, and termination, which essentially represents the reconfiguration of the network depending on needs and emerging issues. The implementation of chained software blocks over general-purpose hardware is commonly referred to in softwarization research as the Service Function Chain (SFC). The importance of dynamic SFC in NFV is highlighted in [51]. This study presents how dynamic chaining enables flexible VNF allocation to meet specific service requirements and mitigate issues arising from cyberattacks. This capability is essential for ensuring the adaptability of network services to changing conditions and user demands. The researchers introduced a mechanism that not only detects but also prevents cyberattacks.

Another trend in NFV research, which includes SFC, is the exploration of blockchain technology. Blockchain has emerged in recent years and is widely accepted as a promising solution for addressing security and privacy issues in softwarized 6G networks [52]. The use of blockchain in NFV can provide a decentralized approach to VNF management, ensuring the integrity and authenticity of services. Researchers in [53] discuss how blockchain can be used for 5G network security, addressing vulnerabilities associated with traditional centralized architectures. This approach highlights the potential of combining multiple advanced technologies to create more resilient network infrastructures.

The concept of automated network management without human intervention (zero-touch network management) is becoming increasingly popular within the NFV community. The goal of this approach is to fully automate the process of VNF deployment and management, reducing the need for manual operations and enabling faster service delivery. The study [54] on capacity allocation for automated network segmentation based on predictions demonstrates how automation can enhance operational efficiency and optimize service provisioning. This approach reflects a broader trend toward self-optimized networks (SON) that automatically adapt to changing conditions without the need for human oversight.

### 2.4. Research on the Implementation of AI in Softwarized Networks

The role of AI in enhancing security within SDN is one of the key aspects of its application in softwarization. The authors of [55] emphasize that AI-based management systems can significantly improve SDN security by enabling proactive threat detection and response mechanisms. By analyzing network traffic in real-time, AI algorithms can identify anomalies that may indicate security breaches, allowing for rapid intervention before significant damage occurs. This capability is particularly important in the context of increasingly sophisticated cyber threats targeting mobile network infrastructure. The application of AI in SDN is also expanding to optimize energy consumption within mobile networks. The authors of [56] demonstrate that AI techniques can be used to predict network configurations that enhance energy efficiency, which is becoming increasingly important in the context of sustainability. Through intelligent energy resource management and reduction in energy waste, AI can contribute to the overall sustainability goals of mobile network operators. The potential of AI to improve service quality in mobile networks is also significant. The study conducted in [57] shows how AI-driven intelligent routing can enhance data transmission efficiency in SDN by adapting routes based on real-time network conditions. This flexibility allows data packets to be transmitted through the fastest available path, reducing latency and improving overall user experience.

Therefore, the latest research in the field of combining AI with network softwarization reflects a dynamic and rapidly evolving area that is already transforming traditional mobile networks. By focusing on security, performance optimization, integration with new technologies, and addressing scalability challenges, the utilization of AI in network softwarization is a concept that is paving the way for more flexible, efficient, and resilient network architectures that are realized by means of network softwarization. As network softwarization in combination with the utilization of AI continues to develop, its potential to revolutionize innovation in network design, management, and security will undoubtedly shape the future of mobile communication networks.

## 3. Technological Foundations of Network Softwarization

### 3.1. Software-Defined Networks

According to the Open Networking Foundation (ONF), a nonprofit organization focused on the development, standardization, and commercial aspects of SDN, SDN represents a modern concept for the realization of network architecture in which network management functions are separated from data forwarding functions, making the network directly programmable [26]. To achieve this separation, the SDN is based on the use of software-based (SDN) controllers for enabling centralized network control and management, and application programming interfaces (APIs) for enabling communication with the hardware infrastructure that includes switches and routers.

**Figure 4:**
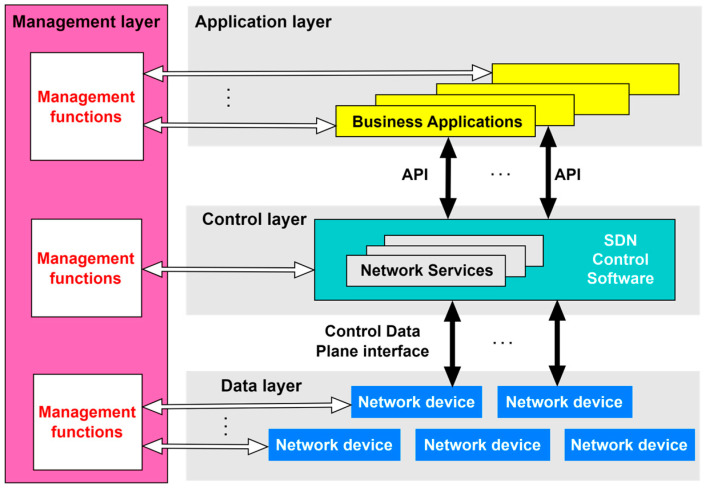
SDN architecture [60,61].

Since network traffic management is conducted through software applications from a centralized location, the implementation of SDN opens possibilities for achieving more flexible and efficient management of mobile networks. SDN also significantly simplifies network devices, as they no longer need to interpret and process a huge number of network protocols and instead, network devices only need to execute instructions from the SDN controller. The intelligence of the network is centralized within SDN controllers, which maintain a global view of the network. According to ONF, the fundamental SDN architecture is structured into three layers (planes): the application layer, the control layer, and the infrastructure (or data) layer. Figure 4 shows a logical representation of the SDN architecture. The interfaces, or APIs that facilitate communication between the SDN controller and SDN applications are called northbound interfaces, while the interfaces that handle communication between the SDN controller and SDN-supported network devices are referred to as control data plane interfaces or southbound interfaces. As stated in [58,59], the southbound interface in SDN architecture belongs to the data plane, while the northbound interface belongs to the control plane.

The SDN controller translates application requests and, based on these requests, controls and manages network elements while providing relevant information to SDN applications. The SDN controller can manage competing application requests for limited network resources in accordance with the defined policy [61].

According to Figure 4, the management layer, which is also traditionally part of conventional networks, must still exist and needs to function across all three planes of the previously described SDN architecture. However, it is crucial to note that the overall significance of the management layer has been drastically reduced in comparison with traditional networks.

Each layer of the SDN architecture has its management functions that provide specific operating instructions to the elements of that layer (Figure 4). At the data plane level of the SDN architecture, management functions are necessary for establishing the connection with the network element and informing the network element about the existence of the SDN controller. In the control plane, management functions are related to configuring policies within the controller that define the scope of control granted to a given SDN application and for monitoring system performance. The application plane (layer) defined in end-user contracts and service level agreements (SLA) is used to configure network management functions [61]. In subsequent sections, each SDN layer (plane) is described in terms of its functionality.

#### 3.1.1. Data Layer

The data plane consists of network equipment such as switches and routers specialized for packet forwarding. However, unlike traditional networks, these are simple forwarding elements without built-in intelligence for making autonomous decisions. The concept of SDN has been based on two key functionality principles of the data plane. The first one is simplicity, which is reflected in the process of packet forwarding at the data plane level, which is one of the fundamental and primary functions of the data plane. The second one is universality, which is reflected in the independence of the SDN architecture from the technology used for network implementation.

**Figure 5:**
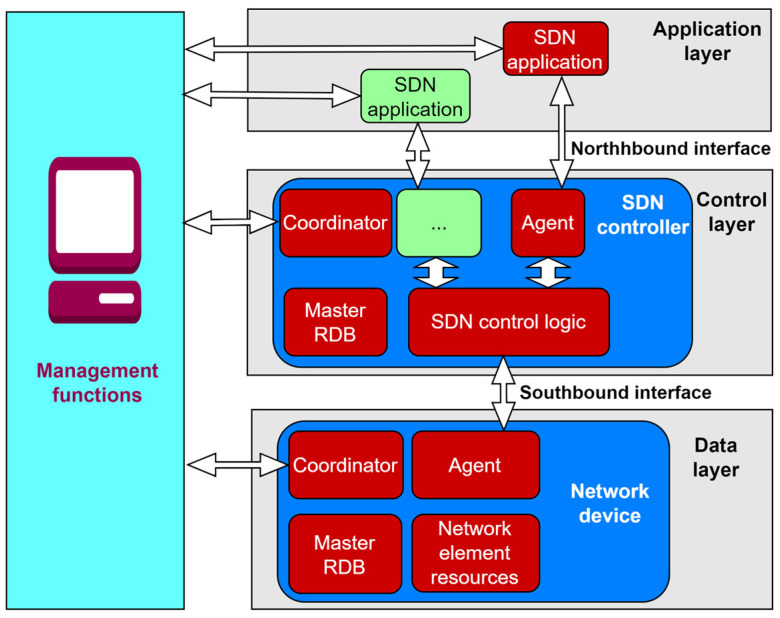
Detailed view of the SDN layers [61].

Therefore, the data plane includes resources that directly manage user traffic, along with the necessary auxiliary resources that ensure proper virtualization capabilities, connectivity, security, availability, and quality [61]. Figure 5 presents a detailed view of the data plane.

According to Figure 5, the data plane contains different entities, such as a master resource database (RDB), that serves as a repository of all resource-related information known to the network device/element. The network element resource block includes information about data input ports, data destinations, and mechanisms for forwarding and/or processing traffic (Figure 5). The control of data forwarding or traffic processing functions can be managed by the SDN controller or by separate mechanisms orchestrated in collaboration with the designated SDN controller. The data plane executes forwarding decisions received from the control plane and does not autonomously make forwarding decisions. However, the control plane can configure the data plane to autonomously respond to events such as network failures or to support functions that need to be executed based on information received through different protocols, such as the Link Layer Discovery Protocol (LLDP), Spanning Tree Protocol (STP), and Bidirectional Forwarding.

Detection (BFD), or Internet Control Message Protocol (ICMP). In addition, the data plane agent is an entity that executes the instructions of the SDN controller within the data plane (Figure 5).

The data plane coordinator is an entity that manages the allocation of data plane resources to various client agents (at higher levels, they can be at the control plane) and defines policies for their use (Figure 5). Agents and coordinators serve the same role at every level of the network architecture [61]. It is important to note that due to the abstract nature of the SDN architecture and its layers, it does not matter whether the lowest level consists of actual dedicated physical or virtual entities, as long as the assigned functions at the data plane level are executed in accordance with the instructions received from the control plane (layer). The protocol called OpenFlow is the most commonly implemented interface between the data and control planes (i.e., the southbound interface). The OpenFlow protocol enables communication between the control and data planes (layers), which is crucial for implementing the concept of network programmability in SDN networks [62]. Through this protocol, hardware supporting the OpenFlow protocol can be used for designing new networks and analyzing network performance. Additionally, the OpenFlow protocol can also be implemented on virtualized network elements. The advantages that MNOs can achieve by using an SDN architecture based on the OpenFlow protocol include: centralized management of network elements in multi-vendor environments, reduced network management complexity through implementation of network management automation, higher possibility of introducing new services, increased network reliability and security, more granular network control, and improved user experience [60].

If the OpenFlow protocol is to be implemented and applied on the southbound interface of the SDN network, it is necessary to have an SDN controller that supports OpenFlow operation, as well as switches, i.e., network elements that are OpenFlow-enabled. In addition to these two requirements, an OpenFlow communication channel is also essential. In further sections, the functionality of the OpenFlow switch and the characteristics of the OpenFlow communication channel are presented.

##### OpenFlow Switch

**Figure 6:**
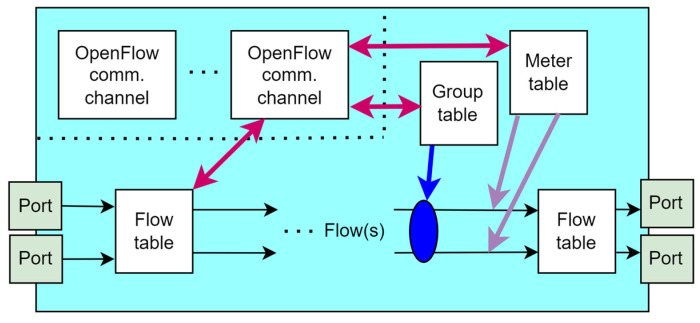
OpenFlow switch architecture [63].

OpenFlow switches are OpenFlow protocol-compatible network devices. Figure 6 presents the architecture of an OpenFlow switch. The operation of OpenFlow switches is based on the three different types of tables. Group table that triggers various types of actions that affect one or more flows, a meter table that triggers various types of performance-related actions on a single flow, and a flow table that specifies the actions to be performed on packets and associates incoming packets with a specific flow (Figure 6) [62].

Instead of routing or media access control (MAC) tables used in traditional switches (operating on the 2nd or 3rd ISO/OSI layer), the flow tables (containing information about various data flows, which essentially represent forwarding rules) enable data forwarding in the OpenFlow switches. The flow table entries primarily consist of match information, specific priorities, counters, and actions that need to be executed. Each packet entering the OpenFlow switch matches the forwarding rule with the highest degree of similarity. The action on the packet, such as forwarding, dropping, or modifying, is executed based on the actions defined in the specified rule, while simultaneously updating the corresponding counters. If a packet does not match any rule in the flow table, the switch can either send the packet to the controller for a decision or simply discard it. When a flow rule expires, the switch must contact the controller to update the flow table.

Equipment manufacturers such as Cisco, Broadcom, International Business Machines Corp (IBM), Juniper, Huawei, and others have introduced commercial OpenFlow switches to meet diverse market demands. These switches are categorized into those designed exclusively for OpenFlow and those that are OpenFlow-compatible. The first category consists of switches specifically developed to support the OpenFlow protocol, ensuring that all packets passing through them adhere to OpenFlow specifications. On the other hand, the goal of OpenFlow-compatible switches is to add OpenFlow-related functionalities to existing commercial switches, facilitating the transition of operators from traditional networks to SDN [64].

OpenFlow ports are network interfaces for transmitting packets between the OpenFlow processing element and the rest of the network (Figure 6). The OpenFlow protocol within SDN architectures can be implemented in three ways: proactive, reactive, and hybrid. In the proactive mode, the controller or network operator predefines and installs the actions that network nodes will apply to traffic flows. However, the proactive application of OpenFlow presents a challenge for most corporate networks, as it requires prior knowledge of all nodes in the network and their possible combinations in future communications to preinstall rules in switches. In the reactive mode, traffic flow management rules are dynamically created in response to changes in network traffic. The hybrid mode combines elements of both proactive and reactive approaches, dynamically adding new flow entries in response to network conditions or potential changes in traffic, when they are correctly detected or predicted [65].

**Figure 7:**
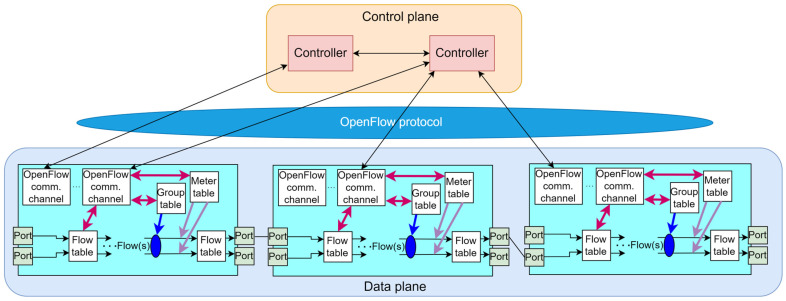
SDN architecture with OpenFlow channels.

The OpenFlow communication channel serves as a link between the control and data planes, enabling mutual data exchange. Figure 7 presents the SDN architecture with OpenFlow different OpenFlow communication channels between the control and data plane. Through this channel, the SDN controller sends instructions and configures the switch, including its activation, authentication, and time synchronization. Conversely, the switch uses the same channel to send the SDN controller information about events it cannot process independently, allowing the SDN controller to make appropriate decisions (Figure 7). All messages transmitted through the OpenFlow channel must comply with the OpenFlow protocol. These channels are most commonly secured using the Transport Layer Security (TLS) protocol, but can also operate based on a Transmission Control Protocol (TCP) connection. The SDN OpenFlow-based controller can manage multiple OpenFlow channels simultaneously (Figure 7), where each channel controls a single switch. Conversely, an OpenFlow switch can also have one or more OpenFlow channels, with each connected to a different SDN OpenFlow-based controller. Typically, the initiator of the OpenFlow channel creation is the switch. However, in specific situations, the SDN controller may initiate the connection, in which case the switch must verify the authorization of that connection [64].

##### Versions OpenFlow Specification

Since the release of the OpenFlow specification version 1.0.0 in 2009, the system has been gradually improved up to the current version 1.5.1, with key differences between versions presented in Table 4. The development of OpenFlow technology has primarily focused on enhancing the performance of devices responsible for data forwarding. This includes support for an increasing number of matching criteria in forwarding decisions, as well as the expansion of flow entries with additional bits for more precise data management. Additionally, a more flexible pipeline with multiple tables has been introduced, enabling faster and more efficient data lookup and comparison. On the other hand, the main limitation of OpenFlow is the limited/fixed set of supported header fields [66].

**Table 4:** OpenFlow protocol versions [64].

OpenFlow Version	Newly Introduced Improvement
1.0	Network segmentation
1.1	Introduction of tables, groups, and virtual ports
1.2	Extensible matching tables, IPv6 support, and multiple controllers support
1.3	Support for per-flow meters, more flexible table-miss, extended IPv6 header, and event filtering by connection
1.4	Support for extensible wire protocol, optical port properties, flow monitor, synchronized tables
1.5	Egress tables, extensible flow entry statistics, TCP flag matching

#### 3.1.2. Control Layer/Plane

The SDN control plane can be realized as the central hub composed of one or more SDN controllers, although control functions in SDN are also partially executed in other planes of the SDN network [61]. The SDN controller is practically the most important element of the control plane. It acts as an intermediary between the application plane, using northbound interfaces, and the data plane, via southbound interfaces (Figure 4). The SDN controller serves a critical network control function as the central management element of the network, providing an abstracted and centralized view of the entire system.

Each control plane contains two main components: applications and the Network Operating System (NOS). Applications can include various software tools, ranging from performance monitoring to network virtualization tools, while the NOS functions as the SDN controller. Properly designed interfaces and a well-configured SDN controller are crucial for the optimal operation of the network. A network may include multiple controllers, with each controlling a specific set of network switches. In such multi-SDN controller configurations, to prevent potential conflicts, one SDN controller is designated as the primary, while the others function as backups [58].

**Figure 8:**
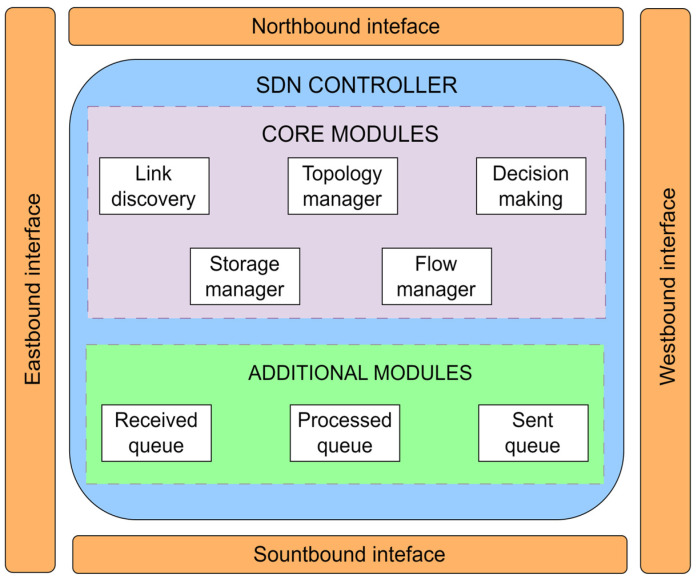
The modular structure of a generic controller [67].

Figure 5 presents the SDN controller on the SDN control layer with a visualization of SDN controller entities. The design of the SDN controller is the most important component of the SDN architecture and significantly impacts overall network performance. Due to this fact, extensive research is being conducted to improve SDN controller design to enhance various aspects of the network, including state consistency, scalability, flexibility, security, availability, latency, and optimal SDN controller placement within the network [38,58]. As shown in Figure 5, the SDN controller contains a coordinator, which is a functional component of the SDN controller that operates on behalf of the functions of the management layer. The agent is an SDN controller entity that, in a specific SDN controller level N, represents the resources and actions available to the client or application of the SDN controller at level N+1. The agent in the data plane at level N−1 represents the resources and actions available to the given SDN controller at level N. The master Resource Database (RDB) element of the SDN controller models the current instance of the network information model and the necessary supporting capabilities. The SDN control logic element (Figure 5) consists of the virtualizer and the Data Plane Control Function (DPCF). The virtualizer processes and validates function requests and translates them into the context of subordinate resources, while the DPCF “owns” and orchestrates these resources through the lower-level agent. Together, they provide functions such as orchestration, resource sharing, event processing, and transactional integrity. Authors in [67] introduced the modular structure of a generic controller, which is presented in Figure 8. 

In addition to the interfaces depicted in Figure 5, this figure illustrates the presence of eastbound and westbound interfaces. These interfaces are designed to facilitate inter-controller communication, enabling coordination and information exchange between multiple controllers within the network when such configurations are present.

The central functions of the network controller are primarily focused on managing network topology and data flows. The operational process of the controller depicted in Figure 8 is initiated by the link discovery module, which periodically generates requests through external ports utilizing *packet_out* messages. Responses to these requests return as *packet_in* messages, enabling the controller to construct the network topology. The topology manager, as a dedicated component (Figure 8), maintains this topology and provides the necessary data to the decision-making module, which is responsible for identifying optimal communication paths between network nodes. These paths are established in a manner that allows the enforcement of specified quality of service (QoS) or security policies during their deployment. Additionally, the controller may include specialized modules such as a statistics collector and a queue manager, responsible for gathering system performance metrics and managing incoming and outgoing packet queues. The flow management module plays a vital role, as it directly interacts with the entries in the flow tables within the data plane [67].

The controller typically operates within a single network subnet, encompassing multiple physical network elements, and does not experience resource conflicts with other entities. This means that the SDN controller assumes ownership of the virtual resources assigned to it through network management. The SDN controller must coordinate multiple interrelated resources, often distributed across multiple subordinate platforms, and sometimes ensure transactional integrity in this process, which is referred to as orchestration. For that reason, the SDN controller is sometimes considered a network orchestrator, but in this case, the need for orchestration within the SDN controller management domain is not eliminated for a lower-level SDN controller with a reduced scope of operation [61].

##### Implementations of the SDN Controller

The standardized SDN architecture does not specify the internal design or implementation of the SDN controller. Table 5 presents the different implementations of SDN controllers [61].

**Table 5:** SDN controller implementation types.

Version	Improvement
Monolithic SDN Controller	A single monolithic process.
Clustered SDN Controller	A set of identical processes that share the load or provide mutual fault protection.
Modular SDN Controller	A series of different functional components that collaborate.
Microservices SDN Controller	A system that utilizes external services for specific functions, such as path computation.

The SDN controller can be considered a ‘black box,’ whose external behavior defines its operation. Its components can operate on various computing platforms, including physical network devices, as well as distributed or virtualized resources such as virtual machines in data centers. The external behavior of the SDN controller is based on the principle of logically centralized control. This principle means that the SDN controller covers the entire global space (within a given context), and its components share information and state among themselves, ensuring that no external entity must independently handle conflicts in the SDN controller’s commands. Multiple management or control components may have shared access to network resources, but they must either manage separate sets of resources/actions or be synchronized to avoid issuing conflicting commands [61].

**Figure 9:**
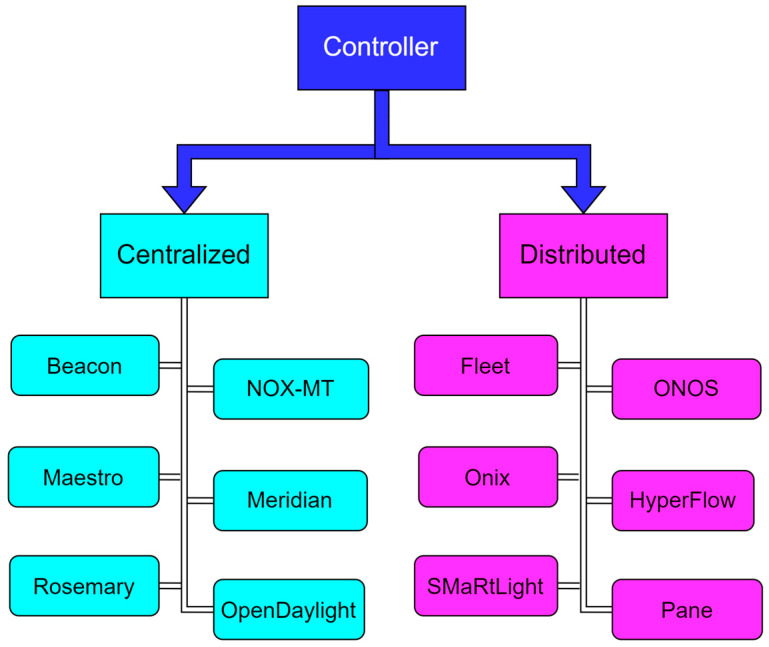
SDN Controller classification [68].

Based on the way the control plane is organized and managed within the SDN architecture, SDN controllers are classified as centralized and distributed. Figure 9 presents the available and popular controllers, categorized according to the previously mentioned classification. Centralized SDN controllers implement the entire control plane logic in a single location. The advantage of this type of controller is the simplicity of management and a global view of the network, while the main realization challenge is scalability, as each controller has a limited data processing capacity.

**Table 6:** Controller type and main features.

Controller	Main Features	Type of SDN Controller
Beacon	High-throughput, Java-based	Monolithic SDN Controller
NOX-MT	Improved version of NOX, multi-threading support	Monolithic SDN Controller
Maestro	Performance optimization through parallelism	Monolithic SDN Controller
ONOS	Large-scale implementations in MNOs, low-latency	Clustered SDN Controller
OpenDaylight	Modular architecture, based on MDSE	Modular SDN Controller
Meridian	Cloud network support, based on Floodlight controller type	Modular SDN Controller
HyperFlow	First distributed SDN controller, uses NOX architecture	Clustered SDN Controller
SMaRtLight	Increased fault tolerance, based on Floodlight	Clustered SDN Controller
Fleet	Protection against malicious administrators	Microservices SDN Controller
ONIX	High scalability, flexible control platform	Clustered SDN Controller
PANE	API for SDN control, conflict resolution among requests	Modular SDN Controller
Rosemary	Micro-NOS architecture, enhanced application security	Microservices SDN Controller

Distributed SDN controllers divide the control logic across multiple controller instances that collaborate with each other. Although this type of SDN controller provides greater scalability and fault tolerance, it requires synchronization between controllers.

Table 6 presents the main characteristics of the centralized and distributed SDN controllers (presented in Figure 9), as well as the SDN classification of controllers per specific controller types (presented in Table 5).

To enable the widespread adoption of SDN architecture, open-source SDN controllers have been developed. The comparative analysis of the key open-source SDN controllers is presented in Table 7 [69,70].

**Table 7:** Comparative view of some SDN controllers [69,70].

Characteristic	NOX/POX	RYU	Floodlight	OpenDaylight	ONOS
Supported languages	C/C++ and Python	Python	Java	Java	Java
Northbound API	Ad hoc	REST	REST, JavaRPC, Quantum	REST, RESTCONF, XMPP, NETCONF	REST, Neutron
Southbound API—OpenFlow Version	1.0	1.0, 1.2, 1.3, 1.4, 1.5, Nicira extensions	1.0, 1.2, 1.3, 1.4	1.0, 1.2, 1.3, 1.4, 1.5	1.0, 1.2, 1.3, 1.4, 1.5
NFV support	Yes	Yes	Yes	Yes	Yes
REST API support	Yes	Yes	Yes	Yes	Yes
Traffic Engineering support	No	Yes	Yes	No	Yes
Load Balancing support	Yes	No	Yes	Yes	Yes
Network Monitoring support	No	No	Yes	Yes	Yes
Web GUI support	No	No	Yes	Yes	Yes
Documentation and learning	Low	Medium	High	High, well-documented	High, well-documented
Topology discovery	Yes	Yes	No	No	Yes
modularity	Low	Non-negligible	Non-negligible	High	High
Multithreading—Parallel Processing	Yes/No	Yes	Yes	Yes	Yes
Consistency support	No	Yes	Yes	Yes	Yes
Network Level	Enterprise	Enterprise	Enterprise	Enterprise	Service Providers (Telcos)

##### Open Network Operating System SDN Controller Implementation

**Figure 10:**
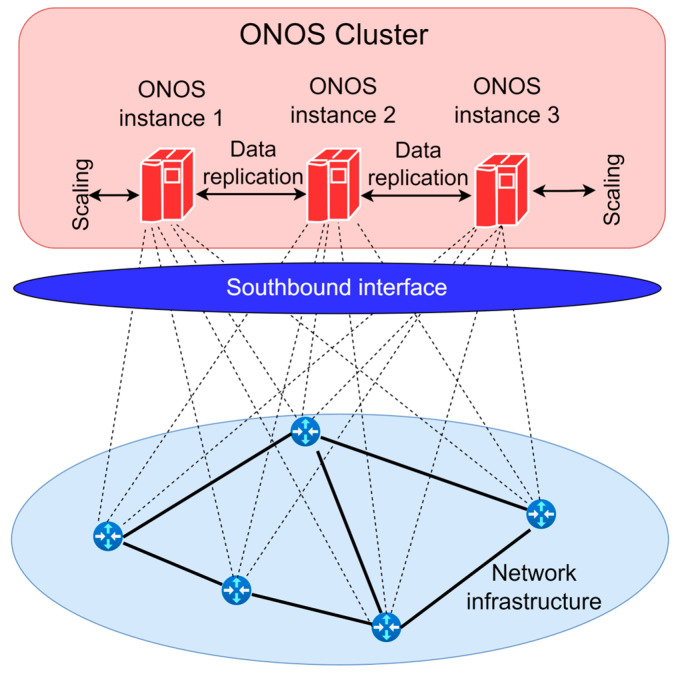
ONOS controller functional architecture.

Open Network Operating System (ONOS) is, in practical implementations, the most preferred among open-source SDN controllers (stated in Table 7), due to several key factors that make it the most suitable for MNOs. ONOS is the first SDN controller designed to meet carrier-grade requirements. Carrier-grade requirements refer to high standards of reliability, availability, performance, and scalability that network systems and solutions intended for telecommunication operators must fulfill. Figure 10 presents the distributed ONOS controller functional architecture. The ONOS controller is designed to support large networks, with a particular focus on resilience and rapid response to network changes. Unlike other SDN controllers that use a centralized approach (Figure 9), ONOS employs a distributed core, enabling it to operate in a clustered environment (Figure 10). ONOS instance clusters ensure high availability and horizontal scalability. This means that network capacity can be increased without disrupting network operations. ONOS can scale horizontally, allowing it to support large-scale networks. Its architecture enables data replication between instances, ensuring fault tolerance and increasing network reliability. ONOS is specifically designed for telecommunications and MNOs, making it more suitable for deployment in large-scale networks compared to other SDN controllers such as OpenDaylight, Floodlight, or Ryu (Table 7).

In addition to OpenFlow, ONOS supports multiple southbound protocols, including Network Configuration Protocol (NETCONF), Simple Network Management Protocol (SNMP), and P4, allowing it to control various network devices and technologies. Furthermore, it is integrated with NFV, enabling advanced network virtualization. ONOS is supported by the Open Networking Lab (ON.Lab) and the Linux Foundation and has a well-developed community that continuously contributes to its development. Its documentation is regularly updated and includes tools for developing new applications, making it easier to integrate into complex network systems [69].

Based on all the previously mentioned aspects, currently, the ONOS SDN controller emerges as the primary choice for MNOs, as it meets their requirements for ensuring high scalability, fault tolerance, support for various protocols, and provides a robust platform for network innovations. Its distributed architecture enables greater resilience and flexibility in managing large networks, making it a more attractive solution compared to other open-source SDN controllers. However, in [71], based on simulations, researchers performed open-source SDN controller comparison analysis for six metrics that include throughput, round-trip time (RTT), latency, jitter, packet loss, and data throughput. Results of analyses show that the RYU SDN controller demonstrated the best results in terms of latency, packet loss, and RTT, while the OpenDaylight SDN controller was the most efficient in terms of throughput and jitter. Hence, among the analyzed SDN controllers presented in Table 7, ONOS exhibited the weakest performance across all measured metrics in this study [71]. Although the ONOS SDN controller type is particularly suitable for large-scale MNO implementations, it is shown that the ONOS SDN controller has limitations in specific performance parameters when compared with other prominent open-source SDN controllers.

#### 3.1.3. Application Layer/Plane

Within the framework of the SDN reference architecture shown in Figure 5, the application layer (plane) enables network applications to interact with the control layer. This layer encompasses diverse software components that articulate high-level service requirements and network intents. These software components are conveyed to the SDN controller via the Northbound Interface (Figure 5), formally designated as the Application-Controller Plane Interface (A-CPI). Rather than engaging directly with the complexities of the underlying infrastructure, applications at the application layer (plane) operate on abstracted views of network resources and states, allowing for the expression of service logic and optimization of network management policies or customer behaviors, without the need for low-level configurations.

An important architectural characteristic of the application plane is its capacity to support multiple levels of abstraction. This allows different applications, ranging from traffic engineering and access control to mobility management, to interact with the SDN controller based on their specific operational granularity. Furthermore, the architecture endorses the hierarchical composition of applications and SDN controllers, thus enabling scalable and modular network orchestration (Figure 5). This structural layering promotes reuse, delegation of responsibilities, and better policy enforcement across the network domain. In operational terms, the application plane contributes significantly to enforcing network operating policies and ensuring consistent behavior across services, particularly in environments involving multiple administrative or trust domains. By isolating the service logic from the physical network and enabling secure, programmable access, it fosters innovation, simplifies the deployment of new functionalities, and supports the dynamic adaptation of the network to shifting users or service demands [61].

### 3.2. Network Function Virtualization

NFV technology forms a fundamental pillar to the softwarization of modern mobile network architectures, as it enables greater flexibility, reduces operating expenditures (OPEX) and capital expenditures (CAPEX), enhances scalability, and accelerates the deployment of network services.

**Figure 11:**
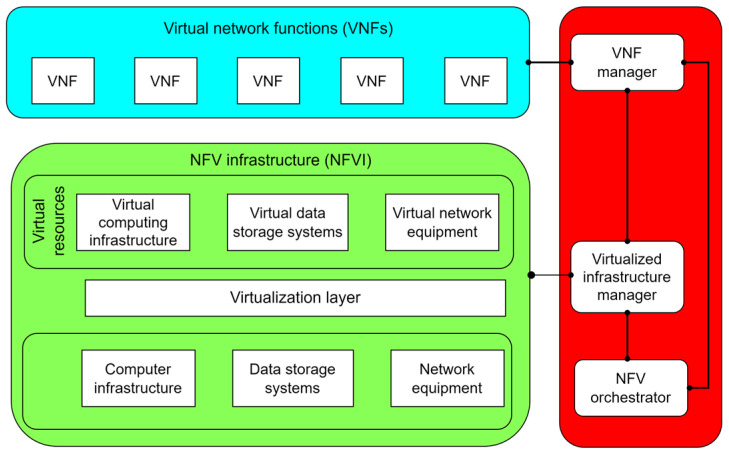
High-level NFV framework [72,73].

A high-level NFV framework is presented in Figure 11. It can be seen that the overall NFV architecture is divided into three fundamental layers: the NFV infrastructure layer, the management and orchestration layer, and the virtual network functions (VNF) layer.

Table 8 presents the key principles that support the NFV architecture and facilitate the aforementioned benefits.

**Table 8:** Key principles of NFV architecture [39].

Key Aspect	Description
Service chaining	NFV technology enables the chaining of multiple VNFs to create complex network services tailored to specific applications and user needs.
Orchestration	NFV relies on an orchestration layer that automates the deployment, scaling, and management of VNFs, allowing network operators to efficiently manage network services without manual intervention.
Standardization	NFV is built on standardized interfaces and protocols that ensure interoperability between different VNFs and NFV implementations, facilitating network service management from multiple vendors.
Flexibility and Scalability	NFV allows MNOs to easily scale network functions as needed, allowing network operators to easily add or remove VNFs based on changes in network traffic, enabling rapid adaptation to changing user demands and market conditions.
Resilience	NFV provides high resilience through built-in redundancy mechanisms and automatic failover to alternative resources in case of failure, ensuring continuous availability of network services.
Multi-tenancy	NFV supports multi-tenancy, enabling multiple users or applications to share the same virtualized infrastructure while maintaining the isolation and security of their network functions.
Reduced Complexity and Automation	NFV relies on automation to simplify the deployment, configuration, and management of VNFs, reducing human error, the need for specialized hardware and skilled personnel and increasing operational efficiency.
Elasticity	NFV enables elastic resource allocation, dynamic scaling VNFs up or down as needed, optimizing resource utilization and reducing operating costs.
Agility	NFV allows MNOs to quickly introduce new network services and innovations, reducing time-to-market and enhancing competitiveness.
Cost Savings	NFV enables network operators to reduce costs by consolidating network functions onto a smaller number of physical devices, thereby lowering hardware and maintenance expenses.

#### 3.2.1. NFV Infrastructure Layer

The NFV Infrastructure (NFVI) layer provides computing, networking, and storage resources necessary for executing VNFs, enabling the replacement of specialized network hardware with commercially available off-the-shelf (COTS) computing infrastructure. In comparison with traditional legacy network architectures, the NFV layer is structured in a way that enables reducing costs and increasing the flexibility of network services. The NFVI consists of three primary sublayers: the physical infrastructure sublayer, the virtualization sublayer, and the virtual infrastructure sublayer.

The physical infrastructure sublayer is related to the hardware resources and includes computing nodes, computing infrastructure/hardware, storage systems, and network equipment. The computing hardware consists of servers with multi-core processors, while data storage is implemented through solutions such as Direct Attached Storage (DAS), Network Attached Storage (NAS), or Storage Area Network (SAN). The network hardware includes standardized ISO layer 2/3 (L2/L3) network devices that support both conventional and SDN-controlled networks, with network interface cards (NICs) being a key component enabling connectivity between VNFs located in different locations.

The virtualization sublayer provides an abstraction of the physical infrastructure through hypervisors and containerization. Certain hypervisor implementations enable the creation and isolation of virtual instances on the same physical hardware. However, alternative hypervisor solutions offer more performance-efficient implementations with reduced resource consumption, which consequently introduce additional challenges related to the security and isolation of virtualized physical infrastructure. The virtualization layer allows for the dynamic allocation of resources among different VNF instances to optimize performance.

The virtual infrastructure sublayer encompasses virtualized computing, networking, and storage resources. In the virtual infrastructure sublayer, software-defined components provide flexibility and scalability for realizing network services, while virtual computing resources enable dynamic resource allocation of VNFs according to network requirements. The virtual network relies on SDN technologies for dynamic traffic management, whereas virtual storage utilizes Software Defined Storage (SDS) solutions to ensure high availability and scalability [73].

Although NFVI can bring significant advantages in the implementation of modern mobile networks, it also faces significant implementation challenges. These challenges are mostly manifested in performance and network latency due to generic hardware implementations, interoperability issues arising from implementing heterogeneous hardware solutions of different vendors, and security threats. Standardization and security policies tend to ensure the resilience of the NFV ecosystem, while some network virtualization technologies, such as Data Plane Development Kit (DPDK) and Single Root I/O Virtualization (SR-IOV), can improve network throughput and reduce latency.

For example, the DPDK technology includes a comprehensive set of libraries and drivers designed primarily to accelerate packet processing across various processor architectures. By bypassing the traditional network stack in the operating system kernel, the DPDK eliminates the need for frequent transitions between the user and the kernel space of operating systems. This results in significantly higher data plane throughput, while also reducing data transmission latency. The SR-IOV technology is a standard that enables a single physical network card to appear as multiple logically separated virtual devices.

This technology allows virtual machines direct access to physical network resources, aiming to reduce network layer overhead and enhance input/output performance in virtualized environments. The physical device is divided into multiple virtual slices, known as virtual functions. In such implementations of the SR-IOV technology, each virtual function is independently assignable to different VNFs, thereby eliminating constraints caused by the limited number of available physical network cards [74].

#### 3.2.2. Virtual Network Functions Layer

According to the ETSI in [75], a VNF is defined as a network function implemented in the form of software running on NFV infrastructure, aiming to replace or complement traditional network functions.

**Figure 12:**
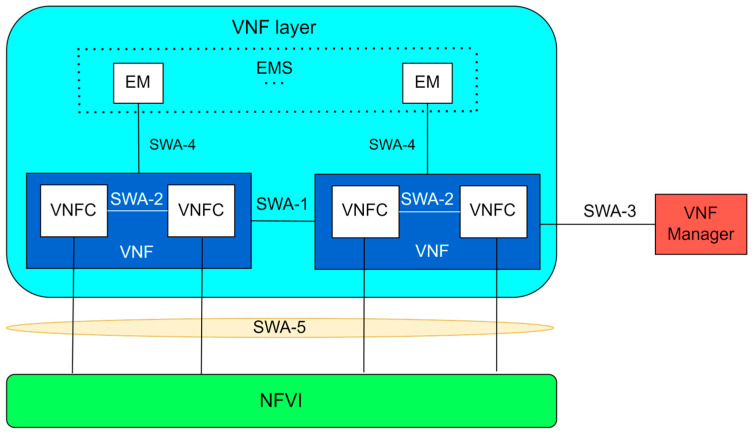
VNF functional view [73,76].

Figure 12 presents the architecture of the VNF layer. According to Figure 12, the VNF layer consists of multiple VNF instances. Each instance comprises virtual network function components (VNFC), which are managed by separate element managers (EMs). The element management system (EMS) coordinates all VNF instances (EMs) and collaborates with the VNF manager (VNFM) located within the management and orchestration layer (Figure 11). The coordination includes processes such as initialization, scaling, and termination of VNFs. The interfaces SWA-1 to SWA-5 (Figure 12) are defined as essential components of the NFV architecture, with the purpose of standardizing communication between various functional entities within the NFV environment [76]. The SWA-1 interface represents the connection between VNF instances, also enabling the linkage between VNFs and physical network functions.

The SWA-2 interface is the internal interface and ensures connectivity between VNFC instances within the same VNF. As it is vendor-specific in nature, it is not accessible to external systems (Figure 12). The SWA-3 interface enables communication between a VNF instance and its corresponding VNFM (Figure 12). This interface supports lifecycle management of the VNF, including instantiation, scaling, migration, and termination, using either layer 2 or IP-based connectivity (in Table 9, this interface is referred to as Ve-Vnfm-vnf).

**Table 9:** Interfaces of the NFV MANO architecture [77].

Interface	Description
Os-Ma-nfvo	Ensures communication between OSS/BSS systems and NFVO, enabling network function orchestration and the provision of resource and performance data. It is crucial for service automation and management.
Or-Vnfm	Enables the exchange of information between NFVO and VNFM. NFVO uses this interface for managing the lifecycle of VNFs, including instantiation, updating, and removing VNF instances.
Ve-Vnfm-em	Connects VNFM with the Element Management (EM) System for monitoring and configuring VNF instances.
Ve-Vnfm-vnf	Facilitates communication between VNFM and VNF to enable configuration, performance management, and fault handling of VNF.
Or-Vi	Enables data exchange between NFVO and VIM. NFVO uses this interface for orchestrating virtualized resources and allocating capacity within NFVI.
Vi-Vnfm	Defines communication between VNFM and VIM. VNFM uses this interface for instantiating and managing virtual resources required for VNF instances.
Nf-Vi	Links the NFVI layer with VIM, enabling management of virtualized and physical resources within NFVI.
Or-Wi	Ensures communication between NFVO and WIM to enable efficient management of network connectivity between different NFVI locations.
Or-Or	Used for exchanging information between NFVO instances in different administrative domains. It enables coordination between multiple orchestrators in distributed NFV environments.

Furthermore, the SWA-4 interface connects the VNF to its EM system and is primarily used for operational monitoring, configuration, and support functionalities within the scope of management models. The SWA-5 interface establishes the connection between the VNF and the NFVI, enabling access to virtualized resources, including computing capacity, memory, storage, and network services (Figure 12).

By replacing physical network elements with virtualized equivalents, the dynamic allocation of VNF instances and changing the serving of different services among network elements becomes possible. This further enables optimizing network performance and energy efficiency.

The implementation of VNFs can be carried out in Virtual Machines (VMs) or within virtualized container-based environments. In virtualized container-based environments, containers provide higher resource utilization density with lower costs; however, container-based implementations also introduce challenges related to security and isolation. Hybrid solutions, such as running containers within VMs or using advanced combined models, allow for the integration of different approaches while addressing potential compatibility and adaptation issues for existing VNFs. The future development of NFV technologies will be focused on optimizing network resources, improving scalability, and further reducing network energy consumption, thus paving the way for more advanced and efficient mobile network management methods [73].

#### 3.2.3. Management and Orchestration Layer

As an important functional layer of NFV systems, the ETSI has defined the management and orchestration (MANO) framework. According to Figure 11, the MANO is a fundamental framework that includes three core functional components: the virtual infrastructure manager (VIM), the virtual network function manager (VNFM), and the NFV orchestrator (NFVO). The VIM is responsible for managing infrastructure resources, the VNFM handles the lifecycle of individual VNFs, while the NFVO coordinates resource allocation and the provisioning of end-to-end network services.

**Figure 13:**
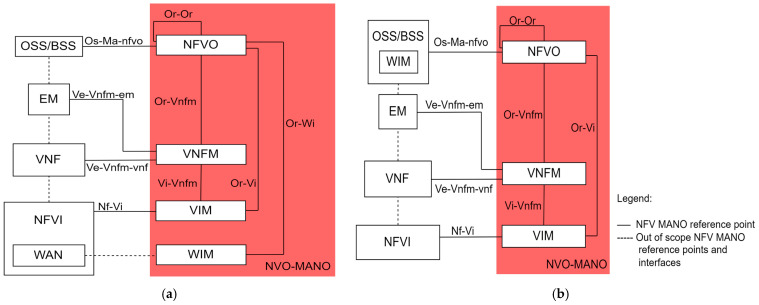
NFV MANO architectural framework: (**a**) with WIM as part of NFV MANO; (**b**) WIM is not part of NFV MANO [77].

In addition to these three functional components, the ETSI in [77] defined a wide area network infrastructure manager (WIM) as a fourth functional component that can be included within the MANO framework. According to document [77] and Figure 13, the implementation of WIM in the MANO architectural framework can be realized in two ways.

Figure 13a illustrates the NFV-MANO architectural framework with WIM as an integrated part of NFV-MANO, while Figure 13b presents the NFV-MANO architectural framework where WIM maintains an external relationship with NFV-MANO. WIM is an entity that manages interconnections between different NFVI Points of Presence (PoPs) through the Wide Area Network (WAN). In the case of direct communication, NFVO communicates with WIM via the Or-Wi interface (Figure 13a) [77].

In the case of ensuing external relationships between operations system support/business system support (OSS/BSS) entities and NFVO, WIM is connected over the Os-Ma-nfvo standardized interface with NFVO (Figure 13b), in order to enable resource coordination and service optimization within the virtualized infrastructure.

In Figure 13, OSS/BSS are the main entities that support operational and business processes within telecommunications networks. OSS includes network resource management, performance monitoring, and fault diagnostics, while BSS encompasses telecommunication processes such as billing, customer management, and service agreements.

Table 9 lists the standardized interfaces of the NFV MANO architectural framework, along with descriptions of their main characteristics. The NFVO is a key component of the NFV-MANO system responsible for orchestrating network functions and managing the lifecycle of network services (Figure 13). The main responsibilities of NFVO include the initialization, scaling, monitoring, and termination of VNF instances, as well as the optimization of resource allocation through interaction with VIM and WIM. NFVO utilizes information from the VNF descriptor (VNFD) and network services descriptor (NSD) to ensure the optimal implementation of network functions within the NFV infrastructure. In this architecture, the descriptor concept refers to the organization of data about VNFs and network services. These descriptors act as centralized data repositories, meaning that all relevant data is stored in a single location to enable easy access, search, and management of network functions and services [77]. The mentioned databases are located within the MANO framework and are directly accessed by NFVO and VNFM [78].

VNFM manages the lifecycle of individual VNFs, including their initialization, configuration, scaling, performance monitoring, and fault recovery (Figure 13). VNFM is responsible for monitoring the state of VNF instances and optimizing them according to network operational requirements. It communicates with NFVO via the Or-Vnfm interface and with VIM via the Vi-Vnfm interface for managing virtualized resources (Figure 13). VIM manages NFVI, including computing, networking, and storage resources. Its primary function is the allocation and optimization of virtualized resources utilized by VNFs. VIM also monitors infrastructure performance and manages faults (Figure 13). It communicates with NFVO via the Or-Vi interface and with the NFVI layer via the Nf-Vi interface.

## 4. The Impact of Softwarization on the Energy Efficiency of Mobile Networks

The aspect of energy consumption in mobile networks is of great importance to MNOs, as it directly affects their OPEX. According to [79], energy consumption accounts for 20–40% of the network operating costs of an MNO. Also, estimates predict that the total energy consumption in the sector of mobile networks could triple over the next ten years, with 5G networks having a significant share in this increase [80]. The increase in energy consumption is particularly characteristic of 5G mobile networks, in which the power consumption per 5G base station (BS) is 68% higher than the power consumption of a multi-radio BS that combines 2G/3G/4G technologies in a single network device [81].

Additionally, taking into account the fact that the implementation of 5G networks requires a significantly higher density of 5G BSs, leads to a key problem that is related to the strong impact of excessive mobile network energy consumption [22,23,24]. This impact is primarily reflected in reduced MNOs’ profitability, and with the massive deployment of 5G mobile networks, this problem will be particularly emphasized in the future.

On the other hand, the introduction of 5G mobile networks leads to a significant increase in data transmission speeds. According to forecasts, the full deployment on the global level of 5G networks is expected by 2030, which is expected to enable a thousand-fold increase in data transmission speeds, while simultaneously halving energy consumption. Therefore, compared to previous generations of mobile networks, it will be necessary to achieve up to 2000 times greater energy efficiency per bit in 5G networks [82].

As stated in the ETSI standard [83], part of mobile network energy efficiency is defined as the ratio of user data to energy consumption of that part of the mobile network. Accordingly, energy efficiency is expressed by the following equation:

(1) $$EE=\frac{UD}{EC}\;\;\;(\mathrm{bit}/J),$$

where EE is the energy efficiency of part of the mobile network, UD is the amount of transferred user data in that part of the mobile network, and EC is the energy consumption utilized for transmission of this amount of data in the analyzed part of the mobile network.

Based on Equation (1), it is evident that to effectively increase mobile network EE in a manner that reduces the OPEX of an MNO, it is necessary to reduce EC or slow down its growth. This can be achieved through different hardware or software-based network approaches that are analyzed in further sections.

### 4.1. Strategies Based on SDN for Improving Network EE

As highlighted in the previous chapters, SDN technology enables centralized network management by separating the control plane from the data plane. Since control data in SDN networks is processed separately from each network element and network elements only perform user data processing based on decisions made by the SDN controller, network elements supporting SDN technology consequently have less need for data processing. The elimination of such control data processing in each network element is directly related to the reduction in energy requirements of each network element, without applying any energy-saving strategies to the SDN network. Thus, the introduction of the SDN concept itself contributes to improving mobile network energy efficiency.

**Figure 14:**
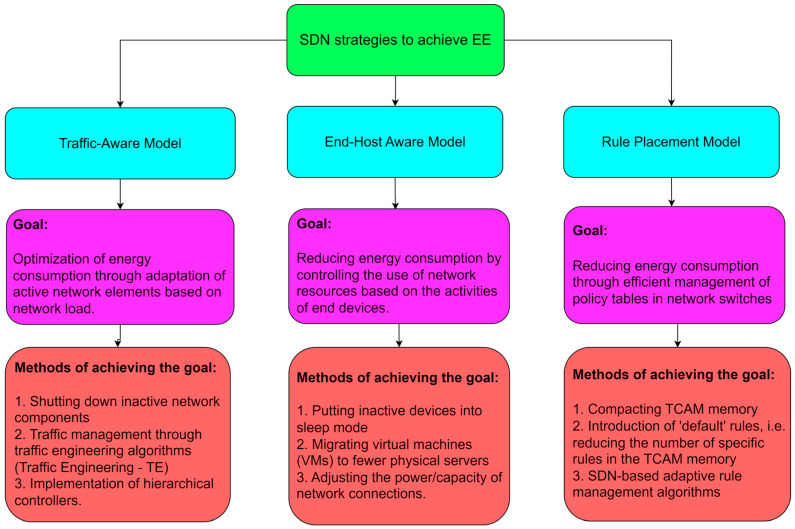
SDN strategies for achieving improved mobile network energy efficiency [84,85].

However, this improvement is far from sufficient to increase the 5G and beyond 5G (B5G) mobile network energy efficiency, which is expected to be significantly more energy efficient in comparison with previous mobile network generations. Therefore, different SDN deployment and operation strategies have been proposed to optimize mobile network energy consumption. According to documents [84,85], three general categories of software-based approaches for optimizing energy consumption have been developed: the traffic-aware model, the end-host-aware model, and the rule placement model. Software-based energy optimization solutions are applied at the SDN controller [85]. These models, along with their basic objectives and methods for achieving mobile network energy consumption optimization, are presented in Figure 14.

The first strategy, presented in Figure 14, is the Traffic-Aware Model. It is based on adapting the activity state of network elements according to network load, including the deactivation of inactive components, the application of traffic engineering algorithms, and the use of hierarchical controllers. The second strategy, the End-Host Aware Model, is oriented toward resource optimization based on the activity levels of end devices, employing mechanisms such as placing inactive devices into sleep mode, migrating virtual machines to a reduced number of physical servers, and dynamically adjusting the capacity of network connections. The third approach, the Rule Placement Model, focuses on optimizing the management of policy tables within network switches, primarily through ternary content-addressable memory (TCAM) compaction, the introduction of generic (“default”) rules, and the implementation of adaptive algorithms based on SDN principles. Each of these strategies contributes to the overall reduction in energy consumption within the network infrastructure, relying on centralized control and programmability enabled by the SDN paradigm.

### 4.2. Implementation of NFV for Improving Network Services Energy Efficiency

**Figure 15:**
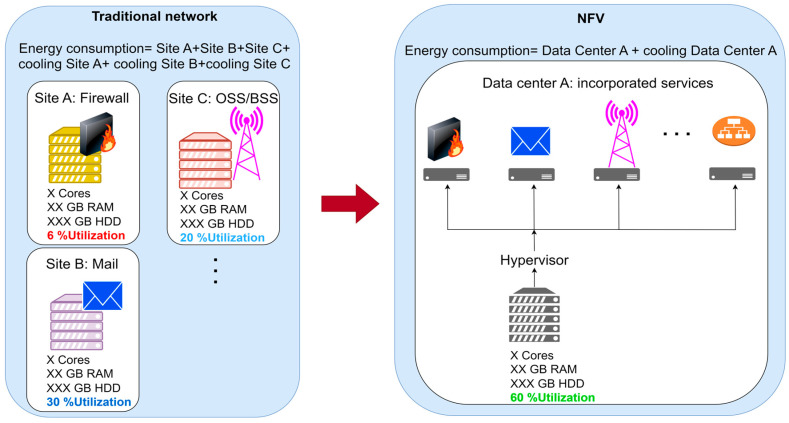
Traditional network vs. NFV concept in the context of energy efficiency.

Instead of the traditional approach to the realization of mobile networks, where each network element executes network functions (services) on individual network elements utilizing specialized hardware, in the NFV concept, network functions can be implemented on standard servers located in data centers. Figure 15 presents the comparison of the traditional network and NFV-based networks in the context of energy efficiency.

According to Figure 15, multiple network functions can be executed on a single server, using the resources of one hardware unit, unlike traditional deployments, where each network function has dedicated hardware, each consuming energy separately. Thus, the introduction of the NFV concept itself contributes to improving mobile network energy efficiency, since joint optimization of data center hardware exploitation and temperature regulation can contribute to reducing mobile network energy consumption (Figure 15).

Furthermore, in the NFV concept, virtual network functions are connected to network elements via virtual links, which also have lower energy consumption requirements compared to the execution of network functions on dedicated hardware with physical connections [31].

It is shown in [86] that NFV provides a great advantage in improving network energy efficiency, with 30% energy consumption reduction through the implementation of the NFV concept in 5G network architecture. In the context of energy consumption management, the NFV-MANO plays a crucial role in automating resource allocation and optimization of mobile network energy consumption (Figure 13). To optimize network energy consumption, it is possible through the NFV-MANO to dynamically scale network functions. This includes adjusting the number of active VNFs according to network demands, activating/deactivating VNFs, migrating VNFs to more energy-efficient network nodes, or applying algorithms based on Artificial Intelligence (AI) and Machine Learning (ML) to predict network load and pre-optimize network resources.

### 4.3. Implementation of NFV and SDN in Improving RAN Energy Efficiency

**Figure 16:**
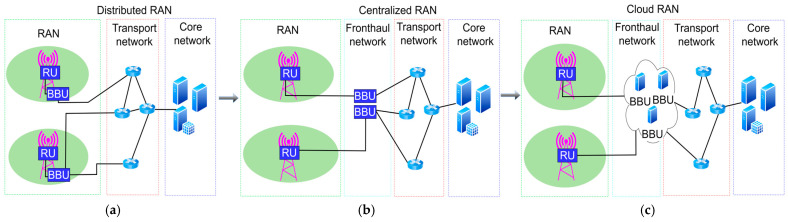
The evolution of RAN architecture: (**a**) distributed RAN, (**b**) centralized RAN, and (**c**) Cloud-RAN [87].

In the context of the implementation of 5G networks, one of the most important applications of NFV is in the development of 5G Radio Access Network (RAN). Figure 16 presents the evolution of RAN architecture, starting from the distributed RAN over centralized RAN to the Cloud-RAN architecture, having mobile network baseband processing centralized and virtualized using NFV. This evolution of mobile network architecture is implemented through the softwarization of a radio access network based on Cloud-RAN (C-RAN) technology. In the C-RAN mobile network architecture, the radio unit (RU) is positioned at the location of BSs, while the Baseband Processing Unit (BBU) functions have been relocated and implemented in data centers through NFV technology.

Such implementation of NFV in RAN enables the reduction in the number of base station (BS) BBUs, through centralization and virtualization of network functions (Figure 16c).

This architecture, based on virtualized BBUs through the implementation of NFV, allows better resource distribution, which consequently results in reduced energy consumption of BSs, representing the largest individual energy consumers in mobile networks [86]. Therefore, this approach to the development of mobile networks contributes to reducing mobile network energy consumption. It is based on the softwarization of the network through the joint implementation of SDN and NFV technologies. Such network softwarization contributes to reducing the energy consumption of BS baseband processing units (BBUs).

Also, it is important to note that in solutions where the BBU is located at the macro BS site, each cabinet in practice additionally needs to have a cooling system to maintain the temperature of the BS equipment, including BBU(s), within the prescribed operational limits. This additionally contributes to the overall increase in mobile network energy consumption. Thus, transferring BBU functions to data centers through softwarization and exploiting SDN and NFV technologies, the overall contribution of BBUs to mobile network energy consumption can be reduced. Also, cooling costs are shared with other services running on the servers of data centers, which results in more economical and efficient energy resource management.

Furthermore, C-RAN implementations of BBUs utilizing SDN and NFV technology can contribute to improving the energy-efficient scheduling of network resources. Applying advanced RAN resource management algorithms in SDN and NFV-based C-RAN architectures is more applicable and straightforward in comparison with traditional networks having separate BBU implementations at the location of each BS site (Figure 16). Such advanced energy management algorithms can, in softwarized networks, perform the scheduling of network resources in the form of activating and deactivating virtual BBUs, according to traffic variations characteristic of a specific RU of some BS. Thus, softwarized networks based on SDN and NFV technologies are particularly suitable for the implementation of such advanced energy-efficient scheduling of network resource activity (e.g., BBUs), which can significantly contribute to improving mobile network energy efficiency. Additionally, data centers hosting virtualized BBUs can use power from renewable energy sources, thereby further improving the overall energy efficiency of the system.

Also, in the context of the dynamic adaptation of RAN hardware resources, the implementation of Dynamic Voltage and Frequency Scaling (DVFS) is seen as an important concept for reducing different RAN devices’ energy consumption during the execution of VNFs. The DVFS technology is based on dynamic adjustment of the voltage and frequency of the electronic components (e.g., processor), in order to optimize their energy consumption based on traffic load variations, while maintaining acceptable network performance. Thus, in the NFV environment, the DVFS concept is primarily applied to reduce energy consumption on processors of network elements responsible for executing virtual network function(s). However, certain NFV services require stable and predictable performance levels, which in some cases impose limitations on the use of DVFS to avoid increased latency or reduced throughput [88].

By utilizing advanced resource management mechanisms and intelligent orchestration of network functions, NFV infrastructure can enable dynamic adaptation of network energy consumption to varying network traffic loads. The ETSI in [89] describes various use cases that enable optimization of energy consumption in NFV systems. The NFV infrastructure allows the reduction in energy consumption by switching off inactive resources or transferring them to low-power operating modes, which are triggered in periods characterized by low network load. The NFV-MANO system continuously collects data on the energy consumption of physical resources such as server nodes and network devices, while simultaneously monitoring the energy needs of virtualized network functions and network services, to allow effective resource prediction and real-time energy consumption optimization. This enables dynamic allocation of network functions based on available energy capacities and operational efficiency, with the ability to allocate and migrate execution of VNFs to more energy-efficient nodes. It also ensures rapid recovery from energy-saving states to active operation, in order to maintain the required quality of service (QoS).

Ultimately, NFV-based approaches for improving network energy efficiency can enable adjusting the usage of computing resources of data centers and edge infrastructure according to traffic load variations. This includes capacity scaling, deactivation of inactive resources, and optimization of load distribution, which can contribute to improving mobile network energy efficiency [89].

### 4.4. Improving EE of 5G Mobile Networks Using SDN and NFV

The previous chapters present strategies and techniques for reducing energy consumption in networks having independent implementation of SDN and NFV technologies. Although these two technologies are complementary, the presented strategies and techniques are applicable independently of each other, i.e., they can be applied in networks where only SDN is implemented without NFV, and vice versa. However, the best synergy effect can be achieved when these two technologies are jointly implemented.

#### 4.4.1. Realization of Network Slicing with SDN and NFCV Technology

**Figure 17:**
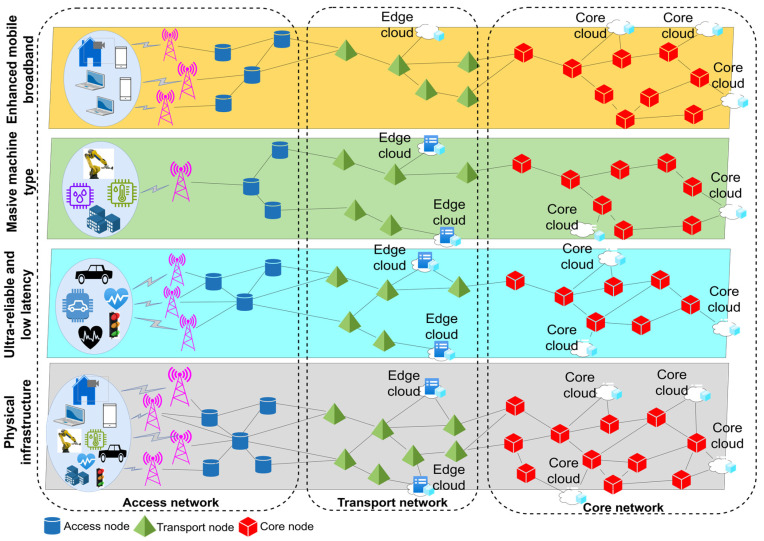
Fifth-generation network slicing architecture [91].

An example of a joint implementation of NFV and SDN in a mobile network is the realization of the network slicing (NS) concept presented in Figure 17. NS is a technology that enables the creation of logically separated virtual networks within the same physical 5G or B5G mobile network infrastructure [90].

In addition to benefits such as improved latency and dedicated throughput to network slices, the application of NS itself contributes to increasing the energy efficiency of 5G networks. This is due to network resources that are precisely tailored to specific service or use case needs, without excessive exploitation of unnecessary network resources.

From Figure 17, it can be observed that there are end-to-end paths in every slice, indicating that a network slice is a logical network capable of operating independently. 

The network slice can also be offered as a service to interested parties, which can on such infrastructure provide their services to users. These services may neither be new nor different in any way from the services offered by the owner of the physical network infrastructure on which such network slice operates. In this way, the interested party can become a mobile operator without owning any infrastructure related to mobile networks. Such a mobile operator is referred to as a mobile virtual network operator (MVNO).

**Figure 18:**
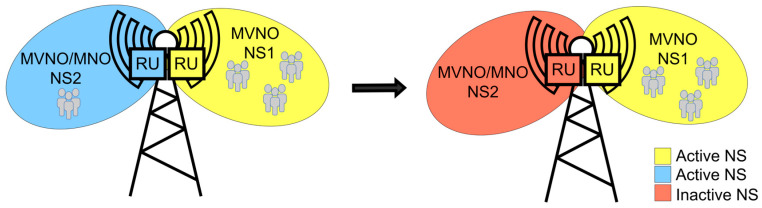
MVNO and MVNO/MNO in the same geographical location.

An example of the implementation of NS with two network slices (NS1 and NS2) in the RAN part of the mobile network is presented in Figure 18, where one NS belongs to the MVNO and the other to the MVNO/MNO operating in the same geographical location.

In the context of energy savings, the existence of two network slices in the same geographical area providing different services can lead to additional energy savings. This can be achieved through the implementation of NS using NFV and SDN technologies, in such a way that when one network slice has no active users, the part of the network elements serving this network slice can be completely put into an idle (energy-saving) state. In this way, the NS that is in the idle state consumes low or no energy at all, while the other network slice(s) remain active [90]. This concept can be implemented in scenarios with multiple network slices that exist in the same geographical location, having time periods with no user device activity in a specific network slice. Therefore, specific network slices in periods of no user activity can be scheduled in a low-power consumption state through combining SDN and NFV-based resource scheduling, which contributes to reducing mobile network energy consumption.

#### 4.4.2. Implementation of AI with SDN and NFCV Technology

In traditional mobile networks lacking the dynamic and programmable layer introduced by SDN and NFV technologies, the application of AI in network resource management usually has a limited possibility for long-term optimization of network energy efficiency. In contrast, mobile networks realized based on an SDN and/or NFV technology can directly embed intelligent AI-based mechanisms into the control and orchestration layer of the network, thereby achieving automated real-time mobile network operation [90]. Moreover, in the operation of fully softwarized 5G networks, it is more convenient to integrate AI algorithms in comparison with traditional mobile networks. Exploiting AI algorithms in scheduling network resources can be a powerful and important approach that can contribute to reducing energy consumption and achieving improved network energy efficiency. In mobile networks, the main focus is on reducing and optimizing energy consumption in the RAN part of the network, as this is the part of mobile networks with the highest energy consumption, accounting for as much as 70% of the system’s total energy consumption [92]. According to [93], the resources of most BSs remain unused for 75–90% of their total operating time, although they may occasionally experience significant traffic loads. Thus, the implementation of AI in networks utilizing SDN and NFV technologies has the potential to significantly improve mobile network energy efficiency through optimized scheduling of BS resources and operating time.

Therefore, the involvement of AI represents a breakthrough in the development of management and orchestration strategies for increasing mobile network energy efficiency, and currently constitutes an additional pillar in the synergistic operation of SDN and NFV technologies [90]. The integration of AI into the mobile network management and orchestration systems opens possibilities for energy savings in spatial, temporal, and frequency domains of the RAN part of the mobile network [31].

**Figure 19:**
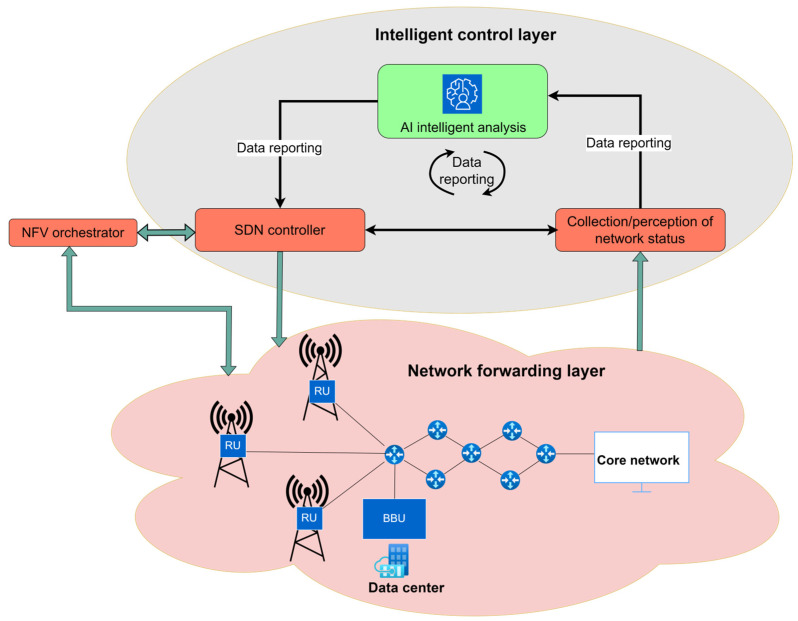
Architecture of intelligent network control [94].

According to Figure 19, in the architecture of a 5G standalone mobile network based on SDN and NFV, the artificial intelligence layer is positioned as the highest layer (above the management and orchestration layer of the SDN controller and the NFV orchestrator). Such a position of AI (Figure 19) enables an AI decision engine (module) in the mobile network architecture to have access to real-time information related to the network traffic, topology, virtualized resources, and service states, thereby establishing a foundation for energy-efficient decision-making. 

Through load prediction mechanisms and intelligent management policies, AI can dynamically adjust traffic routing, instantiate, activate, or deactivate VNFs, and redistribute resources to optimize mobile network energy consumption without compromising the quality of service. The integration of the AI module at the highest layer of the 5G network architecture facilitates synergistic interaction between automated decision-making, service orchestration, and control mechanisms, thereby significantly enhancing the efficiency of network operations. This enhancement is particularly important in the context of the complex demands related to the 5G services and sustainability objectives. Thus, such an advanced 5G network architecture presented in Figure 19 can enable the development of next-generation self-learning and energy-aware networks.

The ITU in document [95] defines a multi-phase energy-saving model of future 5G mobile networks driven by AI, which includes the collection and processing of network data. The model is executed in the SDN/NFV environments via the SDN controller and NFV orchestrator (Figure 19). It enables the identification of network elements operating scenarios, prediction of network load, determination of thresholds and time intervals for the activation of energy-saving functions, implementation of these functions, and feedback-based optimization. Such a structure enables the AI system to adapt energy-saving strategies in real time to specific traffic conditions and network topologies, thereby optimizing energy consumption without compromising QoS. Concrete examples in 5G and B5G mobile networks can include carrier shutdown, transitioning base stations into deep sleep mode, and dynamic redirection of users to more energy-efficient network elements.

The authors in [96] further emphasize that AI can facilitate the deployment of the self-organizing networks (SON) concept, which automates the processes of monitoring, analyzing, and optimizing network operating parameters. By applying machine and deep learning methods (e.g., long short-term memory (LSTM) networks, reinforcement learning, etc.), SON systems become capable of recognizing changes in network patterns, predicting congestion, and timely adjusting network device configurations to maintain energy consumption balance among different network parts. Furthermore, the study highlights that AI enables real-time optimization of power and resource allocation and enhances BS energy efficiency, while minimizing redundancy in data transmission.

In paper [94], the authors presented an architecture of intelligent network control that includes three key modules: a module for the collection and perception of network status, a module for intelligent AI analysis, and an SDN controller (Figure 19). Within the framework of an intelligent network management architecture based on the utilization of SDN technologies and AI, the control process begins with a module responsible for the collection and perception of network status (Figure 19). This module facilitates the acquisition of information on traffic, link quality, and service flows using protocols such as Telemetry, SNMP, and Netconf for traffic data, Netflow for service flow analysis, and the two-way active measurement protocol (TWAMP) for link quality measurements. The collected data are stored in a unified repository accessible to both the SDN controller and the AI-based analysis module. Based on this data, the AI module performs advanced analytics employing methods such as machine learning, deep learning, and reinforcement learning, while enabling flexible integration with various data sources. In addition, the AI module is capable not only of processing and modeling data, but also creating optimization strategies tailored to current network conditions and predicted traffic loads, which can include energy efficiency strategies for improving 5G network EE. The generated strategies are then received by the SDN controller (Figure 19), which, based on network optimization algorithms, precisely defines adjustment targets and implements the corresponding actions via network devices. The described process completes the loop of intelligent network control, where information is continuously collected, analyzed, and transformed into actions aimed at dynamic and efficient network resource management. Such an approach ensures a high level of automation, adaptability, and energy efficiency, which is essential in complex and demanding environments such as 5G networks [94].

##### Implementations of AI in O-RAN

Open Radio Access Network (O-RAN) network architecture represents the next evolutionary step in the development of the RAN. O-RAN refers to an open architectural concept for the radio access segment of mobile networks, aiming to separate hardware and software components within the RAN part of the network to enable interoperability among equipment from different vendors. It advocates for open and standardized interfaces between network elements, through regulatory governance by the O-RAN Alliance, which advocates for open and standardized interfaces between network elements. O-RAN regulatory has gained particular relevance in the context of 5G, where network operators seek, among other goals, to leverage AI and ML-based concepts for advancing and optimizing mobile network operation. In alignment with this vision, the four foundational principles of O-RAN are: network disaggregation that includes intelligent, data-driven network control enabled by RAN Intelligent Controllers (RICs), network virtualization, and usage of open interfaces in the network [97].

**Figure 20:**
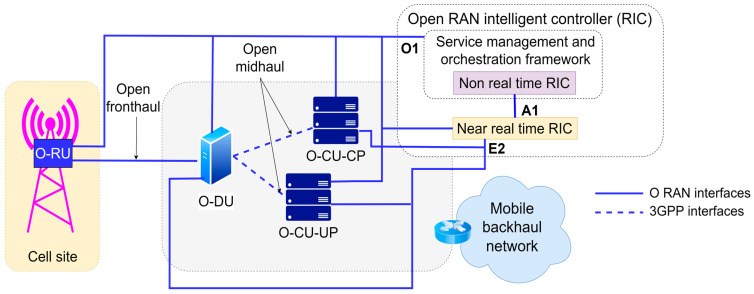
Architecture of O-RAN [97].

The architecture of O-RAN with different data and signaling interfaces (O-RAN and 3GPP) is shown in Figure 20. The open distributed unit (O-DU) represents the distributed or near-real-time processing component within the O-RAN architecture. It is responsible for executing specific protocol layers and processing tasks of the open-radio interface (O-RU). Compared to tasks that are performed by the open central unit (O-CU), these tasks are more closely related to the actual control of the open-radio interface (O-RU). As shown in Figure 20, the O-CU itself is divided into the control plane (O-CU-CP) and the user plane (O-CU-UP). This separation follows the control and user plane separation (CUPS) principle, which facilitates independent scaling and optimization of signaling and data traffic. Such a design allows MNOs to tailor resources according to actual demands: the control plane manages signaling and coordination, while the user plane ensures high-throughput delivery of user data. Additionally, the Open RAN intelligent controller is a component that introduces “intelligence” into the RAN (Figure 20). This is the key element for the realization of energy-saving strategies in the RAN part of the network, since this element contains defined policies for network EE optimization.

The essential interfaces for effective AI integration into the O-RAN infrastructure for improving RAN EE are the A1, O1, and E2 interfaces presented in Figure 20. The E2 interface serves a foundational role in enabling the RIC to manage radio resources and various functionalities of the O-CU and O-DU, as well as to collect network EE metrics from the RAN. These EE metrics can be transmitted either periodically or on an event-driven basis, and need to be based on real data. The E2 interface is defined by two core protocols: the first one is the E2 Application Protocol (E2AP), which manages communication between the RIC and E2 nodes in terms of adopting policies for improving network EE. The second one is the E2 Service Model (E2SM), which specifies specific functionalities such as EE metric reporting and RAN parameter control that will be implemented with the aim of improving network EE. The E2 interface uses the Stream Control Transmission Protocol (SCTP) for the transport of information and supports multiple interaction types, including reporting, inserting, controlling, and policy enforcement, to be a part of the process that contributes to the continuous improvement of network EE. These services allow for the implementation of advanced control and analytic mechanisms within the O-RAN ecosystem, thereby enhancing overall network energy consumption optimization.

The O1 interface links the Service Management and Orchestration (SMO) layer to RAN elements, including the near-real-time (RT) RIC and RAN nodes (Figure 20). Its primary role is to manage the lifecycle of O-RAN components, where initialization, configuration, and performance assurance need to be performed with the aim of optimizing network EE, as well as to support fault monitoring, trace collection, software, and file management. The O1 interface utilizes standardized communication protocols such as Representational State Transfer/Hyper-text Transfer Protocol Secure (REST/HTTPS) and NETCONF. Additionally, it facilitates file transfers, software updates, management of security certificates with beamforming configuration, and deployment of ML models that contribute to improving MNO EE.

The A1 interface links the non-RT RIC with the near-RT RIC, facilitating the transmission of policies dedicated to optimizing network EE and the management of ML models. The non-RT RIC employs A1 to define high-level optimization objectives (e.g., network QoS and EE targets), whereas the near-RT RIC enforces these policies for improving network EE over the RAN. Moreover, A1 supports the exchange of information not inherently available within the RAN (e.g., capacity forecasts) to enhance overall network EE performance.

##### AI Workflow Process in O-RAN

**Figure 21:**
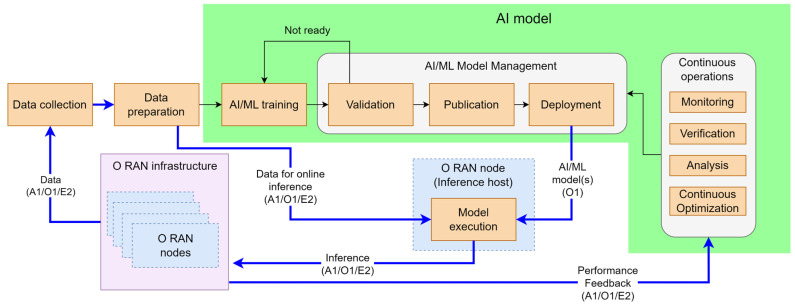
AI/ML workflow in the O-RAN architecture [97].

The non-RT RIC can also serve as an “orchestrator” for energy-efficient AI-based management of network resources, overseeing and managing the entire lifecycle of AI/ML processes that are dedicated to reducing network energy consumption. It consolidates all essential stages in one place, starting from data collection and model training to validation and eventual deployment into the network for continuous operation. The complete AI/ML workflow process in O-RAN is illustrated in Figure 21.

It begins with the collection of all data that are relevant for improving network EE through standardized interfaces (A1, O1, E2). The collected data (such as the number of users on specific BS, number of used channels, subcarriers, or resource blocks, BS capacity usage, levels of signal-to-noise ratios (SNR) or received signal strength indicators (RSSIs), etc.) are aggregated into centralized repositories. This data, relevant for performing EE management of mobile networks, can be collected with varying granularity and key EE performance indicators (KEEPIs). The collected data undergoes through data preparation process. This pre-processing process includes data normalization, reshaping, scaling, and compressing, in order to fit the information structure to the specific input requirements of AI models dedicated to improving network EE (Figure 21). This step ensures data quality and compatibility for both training and real-time inference. Model training is further conducted entirely offline to ensure that only well-prepared, high-performing EE models are deployed in operational networks (Figure 21). It is possible that MNOs experiment with different model architectures and metrics in controlled environments to identify optimal candidates for live deployment of such systems.

Once trained, such models to contribute to improving network EE need to undergo rigorous validations using diverse datasets to assess their performance in dynamic scenarios, such as varying mobile network traffic loads, user densities, and frequency allocations (Figure 21). EE models that fail validation are redesigned and retrained, while successful ones are stored in an AI/ML catalog for operational deployment. Deployment of AI/ML EE models can occur via containerized applications (image-based) or as standalone files (file-based), depending on the execution environment. These EE models are hosted on inference nodes within the O-RAN architecture, and they perform continuous tasks related to improving network EE that can include classification, prediction, or real-time EE policy generation (Figure 21). Outputs of EE models influence the behavior of RAN components via A1 (policy), E2 (control), and O1 (management) interfaces, adhering to strict timing constraints, particularly in near real-time contexts. Once deployed, EE models are subject to continuous operations including monitoring, verification, and performance analysis. This closed-loop feedback system ensures that underperforming EE models can be quickly adjusted or replaced, maintaining optimal network EE performance without service disruption (Figure 21) [97].

The presented processing lifecycle supports EE by enabling AI to intelligently manage network resources. For example, real-time traffic predictions and adaptive policy adjustments can help deactivate underutilized network elements, redistribute user loads, and adjust radio parameters dynamically. By integrating AI into the control loop, O-RAN enables precise, context-aware decisions that can contribute to reducing network energy usage, while preserving network Quality of Service (QoS). Continuous monitoring further allows the system to evolve in response to changing conditions, ensuring long-term network energy-aware optimization.

## 5. Conclusions

The softwarization of mobile networks has become a key factor in shaping modern telecommunication systems, enabling increased flexibility, efficiency, and improved utilization of network resources. By using virtualization and centralized management of network functions, operators can achieve greater network adaptability, thereby enhancing scalability and reducing operational costs. This approach not only optimizes the operation of network systems but also significantly contributes to energy efficiency, which is becoming one of the main objectives in the development of future telecommunication solutions.

Traditional mobile networks often operated based on fixed models of resource allocation, regardless of current traffic demands. Such an approach resulted in increased energy consumption and suboptimal use of infrastructure. With the introduction of software-defined networking and network function virtualization, dynamic resource allocation according to user needs and network traffic has been enabled, resulting in significant optimization of energy consumption. Softwarization allows the network to adapt its architecture in real time, increasing capacity where needed while simultaneously reducing unnecessary resource consumption in low-traffic areas.

One of the essential aspects of modern softwarized networks is their capability to implement artificial intelligence (AI) in the management and orchestration of network resources. AI enables the analysis of network conditions, the prediction of traffic patterns, and the optimization of network function allocation to achieve energy efficiency. The integration of AI models into softwarized networks allows for timely and intelligent decision-making regarding resource allocation, minimizing energy waste while ensuring uninterrupted service quality.

In addition to energy efficiency, softwarization contributes to increased network reliability and resilience by enabling automatic reconfiguration in case of unforeseen disruptions. Furthermore, the use of advanced virtualization mechanisms facilitates modular development of network functions, enhancing system interoperability and scalability.

The future development of softwarized mobile networks should focus on further integration of automated AI-based management solutions, with an emphasis on energy optimization and adaptation of network capacity according to user needs. Moreover, it is important to explore better integration possibilities of softwarized networks with existing and future standards to ensure maximum interoperability and compliance with regulatory frameworks.

In conclusion, the softwarization of mobile networks represents a crucial step in ensuring energy-efficient, flexible, and sustainable communication systems. By utilizing advanced management algorithms, virtualization, and artificial intelligence, mobile operators can optimize their networks, reduce costs, and increase overall infrastructure efficiency. With the continued development and implementation of new technological solutions, it is possible to achieve even greater energy savings and create a sustainable platform for future generations of mobile networks.

## Abbreviations

SDN	Software-Defined Networks
NFV	Network Function Virtualization
ETSI	European Telecommunications Standards Institute
ONF	Open Networking Foundation
ITU	International Telecommunication Union
IoT	Internet of Things
4G	Fourth Generation
5G	Fifth Generation
SA	Standalone
NSA	Non-Standalone
MNO	Mobile network operator
VoIP	Voice over Internet Protocol
SBA	Service-based architecture
AI	Artificial Intelligence
OTT	Over-the-top
CAPEX	Capital Expenditures
OPEX	Operational Expenditures
DDoS	Distributed Denial-of-Service
NS	Network slicing
VNF	Virtual Network Functions
BGWO	Binary Grey Wolf Optimization
DDRL	Double Deep Reinforcement Learning
BIP	Behavior–interaction–priorities
MILP	Mixed integer linear programming
SFC	Service Function Chain
6G	Sixth Generation
API	Application Programming Interfaces
SLA	Service Level Agreements
RDB	Resource Database
LLDP	Link Layer Discovery Protocol
STP	Spanning Tree Protocol
BFD	Bidirectional Forwarding Detection
ICMP	Internet Control Message Protocol
TLS	Transport Layer Security
TCP	Transmission Control Protocol
NOS	Network Operating System
DPCF	Data Plane Control Function
ON.Lab	Open Networking Lab
RTT	Round-trip time
NFVI	NFV Infrastructure
COTS	Commercially available off-the-shelf
DAS	Direct Attached Storage
NAS	Network Attached Storage
SAN	Storage Area Network
NIC	Network interface card
SDS	Software Defined Storage
DPDK	Data Plane Development Kit
SR-IOV	Single Root I/O Virtualization
EM	Element Manager
EMS	Element Management System
VNFM	VNF Manager
VM	Virtual Machine
MANO	Management and Orchestration
VIM	Virtual Infrastructure Manager
NFVO	NFV Orchestrator
WIM	Wide Area Network Infrastructure Manager
OSS/BSS	Operations Support System/Business Support System
NSD	network services descriptor
VNFD	VNF descriptor
PoP	Point of Presence
SNMP	Simple Network Management Protocol
NETCONF	Network Configuration Protocol
3GPP	3rd Generation Partnership Project
IETF	Internet Engineering Task Force
2G	Second Generation
3G	Third Generation
EE	Energy Efficiency
UD	User Data
EC	Energy Consumed
RAN	Radio Access Network
BBU	Baseband Processing Unit
DVFS	Dynamic Voltage and Frequency Scaling
MVNO	Mobile Virtual Network Operator
C-RAN	Cloud-RAN
SON	Self-organizing network
TWAMP	Two-Way Active Measurement Protocol
O-RAN	Open Radio Access Network
ML	Machine Learning
REST/HTTPS	Representational State Transfer/Hyper-text Transfer Protocol Secure
RSSI	Received Signal Strength Indicator
WAN	Wide Area Network
ONOS	Open Network Operating System
LSTM	Long Short-Term Memory
IBM	International Business Machines Corp
RIC	RAN Intelligent Controller
O-DU	Open Distributed Unit
O-RU	Open radio interface
O-CU	Open Central Unit
O-CU-CP	Open Central Unit Control Plane
O-CU-UP	Open Central Unit User Plane
CUPS	Control and User Plane Separation
E2AP	E2 Application Protocol
E2SM	E2 Service Model
SCTP	Stream Control Transmission Protocol
SMO	Service Management and Orchestration
RT	Real Time
KPI	Key Performance Indicator
BS	Base Station
QoS	Quality of Service
